# Bedside POCUS during ward emergencies is associated with improved diagnosis and outcome: an observational, prospective, controlled study

**DOI:** 10.1186/s13054-021-03466-z

**Published:** 2021-01-22

**Authors:** Laurent Zieleskiewicz, Alexandre Lopez, Sami Hraiech, Karine Baumstarck, Bruno Pastene, Mathieu Di Bisceglie, Benjamin Coiffard, Gary Duclos, Alain Boussuges, Xavier Bobbia, Sharon Einav, Laurent Papazian, Marc Leone

**Affiliations:** 1 Assistance Publique Hôpitaux de Marseille, Department of Anesthesiology and Intensive Care, Hôpital Nord, Aix Marseille University, 13015 Marseille, France; 2 Assistance Publique Hôpitaux de Marseille, Service de Médecine Intensive - Réanimation, Hôpital Nord, Aix Marseille University, 13015 Marseille, France; 3 Centre D’Etudes et de Recherches sur les Services de Santé et Qualité, Faculté de Médecine, Aix-Marseille Université, 13005 Marseille, France; 4 Assistance Publique Hôpitaux de Marseille, Service d’Imagerie Médicale, Hôpital Nord, Aix Marseille University, 13015 Marseille, France; 5 Assistance Publique Hôpitaux de Marseille, Service des Explorations Fonctionnelles Respiratoires, Aix Marseille University, 13015 Marseille, France; 6 Center for Cardiovascular and Nutrition Research (C2VN), INSERM, INRA, Aix Marseille Université, 13005 Marseille, France; 7 Intensive Care Unit, Department of Anesthesiology, Emergency and Critical Care Medicine, University Hospital Nîmes, 30000 Nîmes, France; 8 Surgical Intensive Care Unit, Shaare Zedek Medical Center, Faculty of Medicine, Hebrew University, Jerusalem, Israel

**Keywords:** POCUS, In-hospital emergencies, Rapid response team, Handheld ultrasound device

## Abstract

**Background:**

Rapid response teams are intended to improve early diagnosis and intervention in ward patients who develop acute respiratory or circulatory failure. A management protocol including the use of a handheld ultrasound device for immediate point-of-care ultrasound (POCUS) examination at the bedside may improve team performance. The main objective of the study was to assess the impact of implementing such a POCUS-guided management on the proportion of adequate immediate diagnoses in two groups. Secondary endpoints included time to treatment and patient outcomes.

**Methods:**

A prospective, observational, controlled study was conducted in a single university hospital. Two teams alternated every other day for managing in-hospital ward patients developing acute respiratory and/or circulatory failures. Only one of the team used an ultrasound device (POCUS group).

**Results:**

We included 165 patients (POCUS group 83, control group 82). Proportion of adequate immediate diagnoses was 94% in the POCUS group and 80% in the control group (*p* = 0.009). Time to first treatment/intervention was shorter in the POCUS group (15 [10–25] min vs. 34 [15–40] min, *p* < 0.001). In-hospital mortality rates were 17% in the POCUS group and 35% in the control group (*p* = 0.007), but this difference was not confirmed in the propensity score sample (29% vs. 34%, *p* = 0.53).

**Conclusion:**

Our study suggests that protocolized use of a handheld POCUS device at the bedside in the ward may improve the proportion of adequate diagnosis, the time to initial treatment and perhaps also survival of ward patients developing acute respiratory or circulatory failure.

*Clinical Trial Registration* NCT02967809. Registered 18 November 2016, https://clinicaltrials.gov/ct2/show/NCT02967809.

## Background

Patients admitted to conventional hospital wards often develop acute respiratory and/or circulatory failure which requires treatment by a rapid response team (RRT) [1]. Early diagnosis and intervention may prevent further deterioration, resulting in reduced in-hospital mortality [2, 3], yet the outcomes of patients treated by RRT remain highly variable [4]. Previous studies have shown that during emergencies, point-of-care ultrasound (POCUS) facilitates the identification of causes of respiratory and circulatory failure [5, 6]. In observational studies, POCUS seems to improve the likelihood of early diagnosis in such events and decrease the time to administration of treatment [7], including in patients with both acute respiratory and acute circulatory failure [8, 9].

“Handheld ultrasound devices” (HHUD) are the new generation of ultrasound devices. Their diagnostic capabilities have been validated against conventional ultrasound devices for focused echocardiography, lung ultrasound and deep venous thrombosis detection [10–12]. Such devices have the advantage of being easy to use by RRT at the bedside [13], but the clinical value of such use remains unclear.

We hypothesized that introduction of a RRT treatment protocol based on bedside cardiac and pulmonary POCUS findings in the ward would improve diagnosis, time to treatment and outcomes for patients developing acute respiratory and/or circulatory failures in the ward. The primary aim of this study was to assess the impact of implementing such a POCUS-guided management on the proportion of adequate immediate diagnoses (i.e., during the event) in relation to the definitive diagnosis (i.e., upon discharge). The secondary aims were to assess the impact of POCUS on the time to immediate diagnosis, the appropriateness of care, the need for additional diagnostic tests and patient outcomes.

## Methods

### Design

A single-center, prospective, observational, controlled study was performed at the North Hospital of Marseille (Assistance Publique–Hôpitaux Marseille) from November 2016 to November 2018. In accordance with French law [14], patients were informed regarding the use of their data for publication. Because all strategies used in this study were considered standard of care, the study was observational and informed consent was not required. The study was compliance with the Strengthening the Reporting of Observational Studies in Epidemiology (STROBE) recommendations [15].

### Characteristics of participants

During the study period, all consenting adults hospitalized in medical or surgical wards and developing respiratory and/or circulatory failure justifying placement of a call to the RRT were prospectively included. Exclusion criteria were age below 18 years, pregnancy, cardiac arrest, technical limitations to the performance of ultrasound examination in the ward (e.g., surgical dressings, anatomical abnormalities), lung or cardiac transplant, RRT calling for a neurological failure, RRT calling by the emergency department and impossible follow-up.

**Fig. 1:**
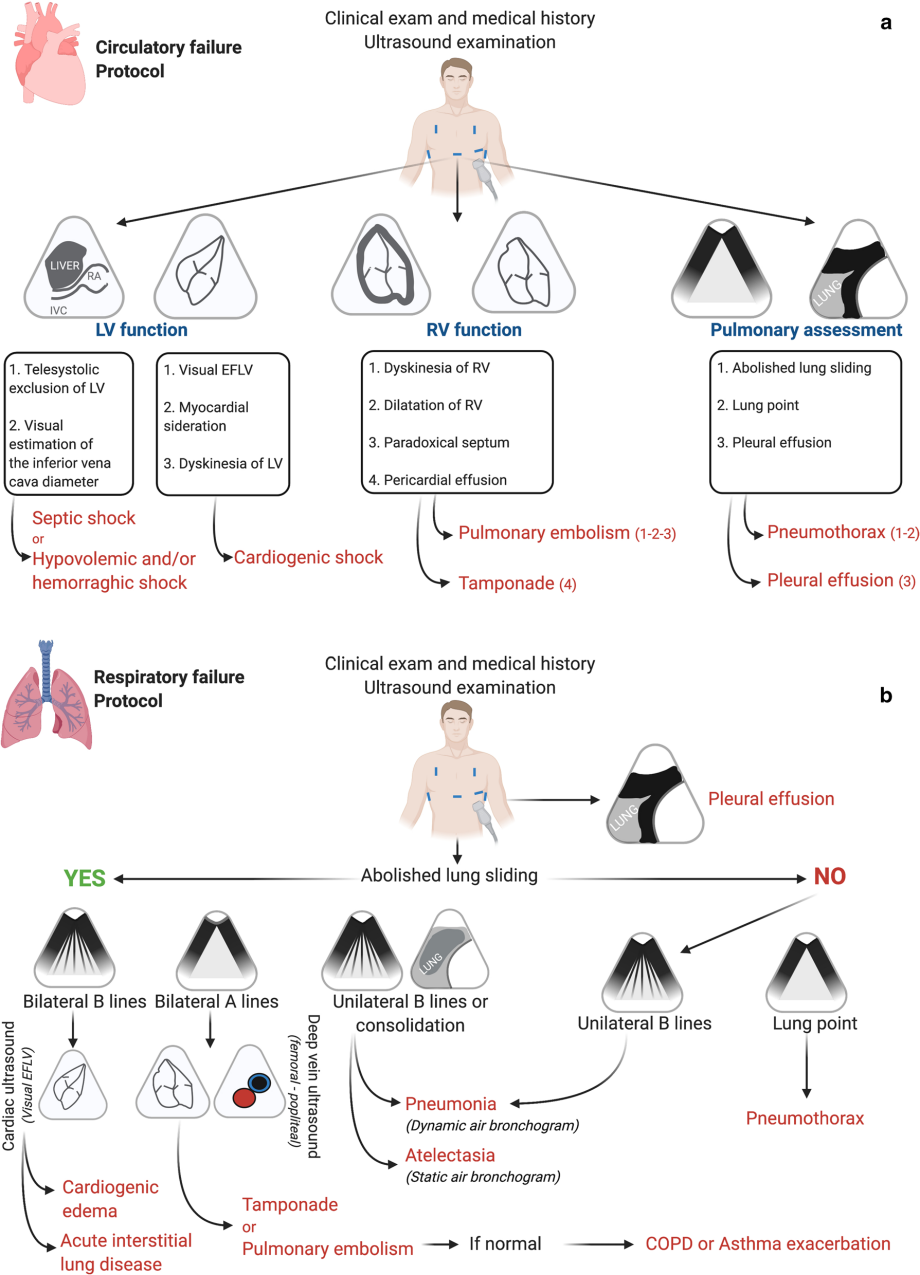
POCUS-guided managements: ultrasound protocol. (**a**) Circulatory failure protocol. Use of the HHUD (Vscan Dual Probe extend^™^, General Electric Healthcare): Cardiac assessment with sector transducer to access the LV and RV function: (1) subcostal view or (2) apical four-chamber view–Pulmonary assessment with the linear transducer: (1) anterior chest based between the clavicle to the diaphragm and the mid-axillary line (2) pleural bases. Abbreviations: EFLV, ejection fraction left ventricular*;* IVC, inferior vena cave; LV, left ventricular; RA, right auricle; RV, right function. (**b**) Respiratory failure protocol. Use of the HHUD (Vscan Dual Probe extend^™^, General Electric Healthcare): Pulmonary assessment with the linear transducer: (1) anterior chest based between the clavicule to the diaphragm and the mid-axillary line (2) pleural bases. Cardiac assessment with the sector transducer to assess the right ventricular function: (1) subcostal view or n apical four-chamber view. Deep venous assessment with the linear transducer to identify thrombosis: femoral and popliteal veins. The visual EFLV helps to differentiate between cardiogenic edema (EFLV altered) and acute interstitial lung disease (non-cardiogenic edema) (EFLV not altered). Abbreviations: EFLV, ejection fraction left ventricular; COPD, chronic obstructive pulmonary disease

In-hospital calls to the RRT were attended by the RRT of two intensive care units (ICUs) in the hospital which alternated every other day. In our institution, the RRT is comprised of a senior ICU physician, an ICU resident and a medical student. The two ICUs cover RRT activity in turns regardless of the study; therefore, a decision was undertaken that one would serve as the POCUS group and the other would serve as the control group (no POCUS using by the control RRT). A protocol for guiding patient treatment based on POCUS findings was developed and made available only to the RRT assigned to the POCUS group (Fig. 1a, b).

### Training and education of physicians

During the three-hour training session (by a level 3 senior physician [16]), typical ultrasound signs of the main causes of acute respiratory and circulatory failure were depicted. This was followed by classroom presentation of practical interactive case reports and a hands-on scenario-based workshop, during which the attendees were required to follow the treatment protocol with a portative ultrasound device. In our department, ultrasound training sessions are offered to both senior and junior physicians twice a year by a level 3 certified senior physician. All senior physicians have at least a level 2 in thoracic ultrasound.

### Study protocol

Calls to the RRT are placed by the physicians in charge of the patients on the ward. Such calls are typically made after an initial assessment by the senior treating physician on location for the presence of organ failures. The reason for RRT intervention (acute respiratory or circulatory failure), the indication for hospital admission and patient features are recorded routinely. Definitions of all terms used within the manuscript are summarized in Additional file 1: Table 1.

#### Clinical assessment

Each patient underwent a standard medical examination by the RRT. This included taking the medical history, performance of a circulatory, respiratory and neurological assessment, monitoring of vital signs, blood testing and conduction of any additional tests judged necessary by the physician in charge. Control patients received treatment according to team assessment based on these data. Study patients also underwent a structured POCUS examination to direct care.

#### POCUS examination in the ward

Patients in the POCUS group underwent an immediate bedside POCUS examination in the ward using a handheld ultrasound device (Vscan Dual Probe Extend™, GE Healthcare). The ultrasound examination included focused cardiac and pulmonary echography and imaging of the deep veins when deemed necessary. The echogenicity of each patient was graded as poor, moderate or good. The examination was interpreted by an ICU senior physician. The POCUS protocol is detailed in Fig. 1a, b [17–20].

#### Immediate diagnosis: management in the ward

Following clinical assessment at the bedside, each RRT selected a diagnosis from a pre-prepared list (the immediate diagnosis). This list was established by the investigators prior to study initiation and validated by the senior physicians involved in the study [7, 9, 21] (Additional file 2: Table 2). In both groups, the time to immediate diagnosis and the time to first treatment were recorded. The number of interventions performed and the number of supplementary tests ordered per patient were collected during review of the medical files for both groups. Each RRT also recorded the treatment the patient received immediately on location and supplemental workup if such was prescribed in the ward (e.g., imaging modalities) as well as patient placement following triage by the RRT (ward, immediate surgery, emergency room or ICU). Patients requiring critical care were admitted to one of the two ICUs according to bed availability.

### Definitive diagnosis

The definitive cause of respiratory and/or circulatory failure was determined following retrospective review of all patient medical files. Two physicians from each team, blinded of the patient group and the initial diagnoses made at the bedside (BP and SH) independently reviewed all the documentation. This included nursing and medical follow-ups, physical examinations and blood and imaging tests. Using this documentation, each determined the definitive cause of deterioration (i.e., the “definitive diagnosis”). In case of disagreement between the two reviewers or if doubt arose, a third expert was consulted (ML) and a consensus was looked for. In several cases, more than one cause was deemed to have led to patient deterioration. In such cases, all of the causes were listed by the reviewing physicians. For determining the percent of adequate diagnosis between the initial diagnosis made at the bedside and the definitive diagnosis, the list of definitive diagnoses reached by the reviewing physicians was compared to the diagnoses documented on the research forms on location of the event. If even one of the diagnoses on the two lists correlated, the diagnosis made at the bedside was classified as correct (Additional file 3: Table 3).

### Sample size considerations

Overall 160 patients were required (80 per group) to detect an absolute difference of 15% between the groups in the proportion of adequate immediate diagnoses in relation to the definitive diagnoses, with an alpha risk and power of 5% and 80%, respectively. This included an assumed 10% dropout.

### Statistical analysis

Baseline patient and treatment characteristics were compared between the control group and the POCUS group. The percent agreement between the immediate diagnoses made at the bedside and the definitive diagnoses (primary outcome) was compared between groups using the *χ*2 test. Comparisons of secondary outcomes were performed using the *χ*2 or Fischer’s exact tests for qualitative variables, and the Student T or Mann–Whitney tests for quantitative variables based on variable distribution.

As sensitivity analysis, multivariate analysis was performed using logistic regressions (enter method) to determine the adjusted effect of the treatment group on in-hospital mortality. Variables were selected for model inclusion if they were of clinical interest and/or if they had a threshold *p* value < 0.1 in univariate analysis. The final models are presented with the odds ratios (ORs) and 95% confidence intervals (95% CIs). A predefined subgroup analysis was performed according to the indication for calling the RRT (respiratory vs. circulatory). Statistical analyses were performed using SPSS (IBM SPSS Statistics version 20 for windows).

To reduce selection bias, we performed a propensity score matching. Eight covariates were included in the propensity score model (Simplified Acute Physiology Score (SAPS) II, mottling’s presence, respiratory rate, pulse oximetry, need for oxygen therapy, chronic heart failure, chronic obstructive pulmonary disease, and sex). The matching was performed using the R MatchIt package and was based on the nearest neighbor with caliper matching (compiled with version 3.5.3; Feather Spray Copyright (C) 2018 The R Foundation for Statistical Computing). Accuracy of the score was assessed using histograms of propensity scores before and after matching and standardized mean differences (SMD) of the covariates. The matching identified 42 POCUS and 73 control individuals. All the details of the propensity scores are provided in Additional file 4: Additional Method.

No data imputation was performed. All the tests were two-sided. Statistical significance was defined as *p* < 0.05.

## Results

**Fig. 2:**
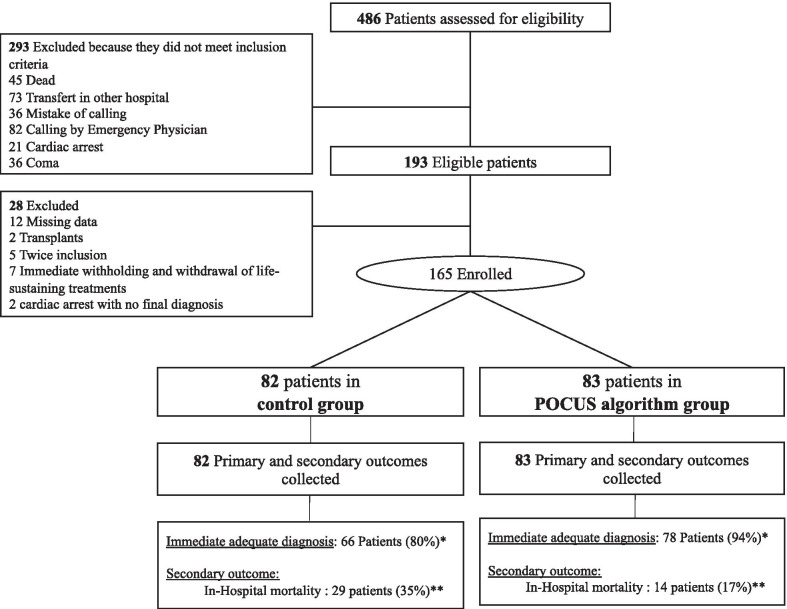
Flowchart. * *p* = 0.009, ** *p* = 0.007. Details of excluded patients by groups: *control group:* 4 immediate withholding and withdrawal of life-sustaining treatments; 2 twice inclusion; 4 missing data; 2 transplant’s patients; 1 cardiac arrest. *POCUS group:* 3 immediate withholding and withdrawal of life-sustaining treatments; 8 missing data; 3 twice inclusion; 1 cardiac arrest

During the study period, 486 patients were screened. Among them, 165 patients were enrolled: 83 to the POCUS group and 82 to the control group (Fig. 2). The patients enrolled in the two groups differed only in the rate of mottling (10% in the POCUS group versus 27% in the control group, respectively, *p* = 0.02), the median oxygen flow rates (6, IQR [2–15] versus 12, IQR [5–15] l/min, respectively, *p* = 0.003) and mean arterial pressure (MAP) (93, IQR [72–113] versus 82, IQR [70–100] mmHg, respectively, *p* = 0.02). The indications for calling the RRT were similar in the two groups (Table 1). In both groups, several cases retained more than one final diagnosis even after three reviewing rounds. The definitive diagnoses retained were similar in the two groups. Senior and junior physicians of the two teams shared the same level of expertise (Additional file 5: Table 4).

**Table 1:** Baseline participants characteristics^a^

Variables	Control group *n* = 82	POCUS group* n* = 83	*P* value
Men	48 (59)	55 (66)	0.31
Age, median [IQR] years	67 [57–77]	70 [59–81]	0.49
*Indication for placing the call to the RRTs*			
Respiratory	56 (68)	61 (74)	0.46
Circulatory	26 (32)	22 (26)	
*Indication for hospital admission*			
Cardiac	10 (12)	3 (4)	0.27
Respiratory	30 (37)	23 (28)	
Neurologic	2 (2)	7 (9)	
Digestive	8 (10)	8 (10)	
Obstetrical	1 (1,5)	0	
Infectious	2 (2,5)	2 (2)	
General	4 (5)	4 (5)	
Surgery	24 (29)	28 (34)	
Urologic	0	2 (2)	
Vascular	1 (1)	3 (4)	
Traumatic	0	3 (4)	
*BMI (> 25), kg/m*^*2*^			
	41 (50)	39 (47)	0.70
ASA score			
I-II	23 (28)	23 (28)	0.99
III	47 (57)	48 (58)	
IV–V	12 (15)	12 (14)	
*SAPS II*^*b*^*, median [IQR]*			
	46 [34–57]	40 [33–52]	0.51
*Comorbidities*			
Allergy	9 (11)	8 (10)	0.78
Coronary disease	22 (27)	23 (28)	0.89
Heart failure	19 (23)	27 (32)	0.18
Chronic kidney disease	11 (13)	19 (22)	0.16
Cancer	28 (34)	26 (31)	0.70
Immunodepression	10 (12)	13 (15)	0.52
Dementia	5 (6)	3 (4)	0.49
Stroke	8 (9,8)	9 (11)	0.82
Epilepsy	5 (6)	1 (1)	0.12
Diabetes	13 (16)	21 (25)	0.13
Liver disease	6 (7)	6 (7)	0.98
COPD	36 (44)	30 (36)	0.31
Habitual smoker	42 (51)	34 (41)	0.19
*Definitive diagnosis*^*c*^			
Exacerbation of asthma	3 (5)	3 (5)	0.60
Septic shock	14 (17)		14 (17)
Pneumonia	18 (22)	7 (8)	
Cardiogenic edema	10 (12)	13 (16)	
Hemorrhagic shock	7 (8)	4 (5)	
Atelectasis	1 (1)	4 (5)	
Exacerbation of COPD	5 (6)	6 (7)	
Pneumothorax	1 (1)	1 (1)	
Pleural effusion	3 (5)	4 (5)	
Acute interstitial lung disease (non-cardiogenic edema)	4 (5)	7 (8)	
Pulmonary embolism	2 (2)	2 (2)	
Cardiogenic shock	2 (2)	2 (2)	
Hypovolemic shock	1 (1)	1 (1)	
Tamponade	1 (1)	2 (2)	
Exacerbation of COPD/cardiogenic edema	0	1 (1)	
Pneumonia/cardiogenic edema	0	1 (1)	
Pneumonia/acute interstitial lung disease (non-cardiogenic edema)	1 (1)	3 (5)	
Exacerbation of COPD/pneumothorax	2 (2)	0	
Normal	0	2 (2)	
Other†	7 (9)	6 (8)	
*Urine volume 24 h before inclusion (ml/day)*			
< 200	6 (7)	4 (5)	0.58
< 500	29 (35)	24 (29)	
> 500	37 (45)	40 (48)	
> 1000	10 (12)	15 (18)	
Mottling^d^	22 (27)	10 (12)	0.02
Respiratory distress syndrome	49 (60)	54 (65)	0.48
Abnormal chest auscultation^e^	62 (76)	59 (71)	0.51
Thorax X-ray < 24 h	35 (42)	38 (46)	0.69
MAP, median [IQR], mmHg	82 [70–100]	93 [72–113]	0.02
Heart rate, median [IQR], bpm*	100 [80–120]	107 [85–130]	0.46
Serum lactate median [IQR], mmol/l	2 [1–3]	2 [1–3]	0.95
Hemoglobin, median [IQR], g/dl	11 [9–13]	11 [9–12]	0.18
Serum creatinine, median [IQR],mmol/L	79 [52–125]	82 [62 -132]	0.51
Total bilirubin, median [IQR] umol/l	10 [8–16]	10 [8–16]	0.91
Serum potassium, median [IQR], mmol/L	4 [4, 5]	4 [4, 5]	0.41
Volemic expansion at the bedside, median [IQR], ml/kg	3 [0–10]	4 [0 -12]	0.73
Respiratory rate, median [IQR] bpm**	27 [20–35]	25 [20–35]	0.51
SpO2, median [IQR], %	92 [89–96]	94 [90–97]	0.16
PaO2/FiO2 ratio, median [IQR]	186 [121–284]	203 [136–355]	0.28
Glasgow score, median [IQR]	15 [2–15]	15 [14, 15]	0.08
BNP, median [IQR] pg/ml	306 [129–584]	188 [62–833]	0.61
Oxygen rate flow, median [IQR], L/min	12 [5–15]	6 [2–15]	**0.003**
*Orientation after inclusion*			
Immediate ICU	59 (72)	49 (55)	0.15
Operative room	5 (6)	7 (8)	
Emergency room	4 (5)	9 (12)	
Ward	14 (17)	21 (25)	

BMI, body mass index; ASA, Score of American Society of Anesthesiologists; SAPS II, Simplified Acute Physiology Score II; COPD, chronic obstructive pulmonary disease; MAP, median arterial pressure; SpO2, pulse oximetry; PaO2/FiO2 ratio, ratio of arterial oxygen partial to fractional inspired oxygen; BNP, brain natriuretic peptide; bpm*: beats per minute; bpm**: breaths per minute; IQR, interquartile range; RRT, rapid response team

^a^Data are expressed as No (%) of participants unless otherwise indicated. At the bedside

^b^The SAPS II ranges from 0 to 163, with higher scores indicating higher risk of mortality. A patient with a score of 40 has an estimated mortality risk of 30%

^c^Diagnosis established after medical files and supplementary workup

^d^Only presence or absence of mottlings

^e^Abnormal was defined like pathological auscultation

^†^Panic attack (1), anaphylaxia (2), bronchospasm (1), hemoptysis (1), intraalveolar hemorrhage (1), laryngeal dyspnea (1), morphine surdosage (1), pulmonary hypertension crisis (1), tracheal decannulation (1), neurological failure (1) and valve thrombosis (1)

### Primary outcome

The proportion of immediate adequate diagnosis at the bedside in the ward was 94% in the POCUS group and 80% in the control group (*p* = 0.009) (Table 2). In the matched cohort, the proportion of immediate adequate diagnosis was 93% in the POCUS group and 78% in the control group (*p* = 0.04) (Additional file 5: Table 5A).

**Table 2:** Primary and secondary outcomes of patients^a^

Outcome category	Control group *n* = 82	POCUS group *n* = 83	P value
Primary outcome			
Immediate adequate diagnosis			
General	66 (80)	78 (94)	0.009
Circulatory	24/26 (92)	21/22 (95)	0.65
Respiratory	42/56 (75)	57/61 (93)	0.006
Secondary outcomes			
Appropriate intervention in ward			
General	72 (88)	77 (93)	0.28
Circulatory	22/26 (84)	20/21 (95)	0.36
Respiratory	48/56 (86)	57/62 (92)	0.28
Time to immediate diagnosis, median [IQR], min			
General	11 [5–20]	10 [5–15]	0.16
Circulatory	14 [7–20]	10 [6–20]	0.94
Respiratory	16 [5–20]	10 [3–30]	0.13
Time to first treatment/intervention, median [IQR], min			
General	34 [15–40]	15 [10–25]	0.00003
Circulatory	22 [14–45]	15 [11–28]	0.26
Respiratory	30 [15–38]	15 [20–61]	0.00003
Number of interventions, median [IQR], min			
General	4 [3, 4]	3 [2–4]	0.0009
Circulatory	4 [3, 4]	3 [2–4]	0.07
Respiratory	4 [3, 4]	3 [2–4]	0.005
Number of supplementary examinations during first day, median [IQR]			
General	2 [1–3]	1 [1. 2]	0.00002
Circulatory	2 [1–3]	2 [1, 2]	0.009
Respiratory	2 [2, 3]	1 [1, 2]	0.0006
ICU length of stay, median [IQR], days			
General	5 [3–10]	3 [2–7]	0.01
Circulatory	4 [2–6]	2 [1–7]	0.23
Respiratory	5 [3–13]	3 [2–7]	0.01
Hospital length of stay, median [IQR], days			
General	16 [9–25]	16 [9–28]	0.44
Circulatory	13 [8–31]	10 [6–29]	0.35
Respiratory	18 [9–29]	17 [10–28]	0.72

IQR: interquartile range

^a^Data are expressed as no. (%) of participants unless otherwise indicated

### Secondary outcomes

POCUS echogenicity was rated poor for cardiac assessment in 9% of cases and for lung assessment in 11% of cases.

The time to immediate diagnosis at the bedside was similar in the two groups. However, the median time to first treatment/intervention was 15 min [IQR 10–25] in the POCUS group and 34 min [IQR 15–40] in the control group (*p* < 0.001). This finding was most pronounced in the subgroup of patients with acute respiratory failure (*p* < 0.001). The proportion of appropriate intervention at the bedside was similar in the two groups. The POCUS group required less supplemental workup than the control group (one additional test [IQR 1–2] versus two additional tests [IQR 1–3], *p* < 0.001) (Table 2).

The rate of patient triage to ICU admission was 55% in the POCUS group and 72% in the control group (*p* = 0.15) (Table 3). For those patients admitted to the ICU, the median length of ICU stay was 3 days [IQR 2–7] in the POCUS group and 5 days [IQR 3–10] days in the control group (*p* = 0.01). The median hospital length of stay did not differ in the POCUS group (16 days [IQR 9–25] and in the control group (16 days [IQR 9–28] days, *p* = 0.44)) (Table 2). All the details of the propensity scores are provided in Additional file 5: Table 5A.

**Table 3:** Administrated treatments by the rapid response teams at the bedside in POCUS group versus the physician judgment (control) treatment groups^a^

	Control group *n* = 82	POCUS group *n* = 83	*P* value
Drainage	8 (10)	14 (17)	0.18
Volemic expansion	40 (49)	29 (35)	0.07
Diuretics	13 (16)	16 (19)	0.56
Nitrates	2 (2)	7 (8)	0.16
Noninvasive ventilation	15 (18)	21 (25)	0.27
Oxygen therapy	58 (71)	53 (64)	0.34
Invasive ventilation	20 (24)	9 (11)	0.02
Aerosol bronchodilators	22 (27)	21 (25)	0.82
Aerosol corticoids	9 (11)	4 (5)	0.14
Systemic corticoid	13 (16)	4 (5)	0.02
Antibiotic	43 (52)	28 (34)	0.02
Curative anticoagulation	4 (5)	5 (6)	1
Aspirin	2 (2)	0	0.25
Inotrope	3 (4)	3 (4)	1
Vasopressor	29 (35)	7 (8)	0.00002
Antiarrhythmic	1 (1)	1 (1)	1
Transfusion	7 (9)	3 (4)	0.21
Physiotherapy	7 (9)	9 (11)	0.61

^a^Data are expressed as no. (%) of participants

The median number of interventions performed per case during bedside management in ward was three [IQR 2–4] in the POCUS group and four [IQR 3–4] in the control group (*p* < 0.001). Patients in the POCUS group also received less invasive mechanical ventilation (*p* = 0.02), systemic steroids (*p* = 0.02) and vasopressors (*p* < 0.001) than patients in the control group (Table 3). All the details of the propensity scores are provided in Additional file 5: Table 5B.

ICU mortality rates were 11% in the POCUS group and 25% in the control group (*p* = 0.04). In-hospital mortality rates were 17% in the POCUS group and 35% in the control group (*p* = 0.007) (Table 4). These differences were not found in the propensity cohort (*p* = 0.79 and *p* = 0.53, respectively) (Additional file 5: Table 5C). The POCUS effect on in-hospital mortality was assessed after adjustment for the main confounding factors: The effect remains significant for most models (sensitivity analyses, Additional file 7: Table 6).

**Table 4:** In-hospital and ICU mortality rates in POCUS group versus control group^a^

	Control group *n* = 82	POCUS group *n* = 83	*P* value
*In-ICU mortality*			
General	* 17 (25)	* 7 (11)	0.04
Circulatory	7/23 (30)	1/17 (6)	0.11
Respiratory	10/44 (23)	6/45 (13)	0.25
*In-hospital mortality*			
General	29 (35)	14 (17)	0.007
Circulatory	12/26 (46)	3/22 (14)	0.02
Respiratory	17/56 (30)	11/61 (18)	0.12

^*^Patients admitted in ICU: control group *n* = 67 and POCUS group *n* = 62

^a^Data are expressed as no. (%) of participants

## Discussion

In this single-center prospective, controlled trial, patients for whom the RRT was called underwent ward treatment that was allocated to be either POCUS-guided management directed or physician judgment directed. Patients in the POCUS group had a higher proportion of adequate immediate diagnosis, were treated more rapidly and had higher survival rates than patients treated conventionally.

A seminal study showed the potential diagnostic impact of lung ultrasound in a cohort of 260 patients admitted to the ICU for acute respiratory failure [18]. Since then, POCUS has repeatedly been proven superior to the combination of physical examination and chest X-ray for diagnosis of patients with acute respiratory failure [5, 9, 23, 24]. In patients with acute circulatory failure, POCUS has a high diagnostic accuracy [6, 25] and can change therapeutic management [26–28]. Despite the major differences between the ICU and the emergency department in terms of staff training and resources, similar results are consistently found in both with regards to POCUS [9, 23, 25]. Our study focused on patients hospitalized in conventional wards where resources are often limited, thereby demonstrating the advantage of POCUS directed treatment in an entirely different environment.

POCUS can be performed by the clinician at the bedside using a handheld device. Compared to other potentially relevant tests, it does not require that a device be brought by another person to the bedside, it is independent of external services and it is noninvasive. POCUS has been described as the fifth pillar of physical examination [29] because it provides more physiological information than do clinical examination, blood gases and electrocardiography combined [30].

Improved diagnostic performance is an explanation for the earlier treatment and reduced number of supplementary examinations observed in the POCUS group when compared with the control group. During emergencies, targeted POCUS screening can include assessment of cardiac function, filling and tamponade, gross valvular function, venous filling, pleural sliding and fluid accumulation as well as deep venous return [10, 31–33]. If a diagnosis is made using POCUS, on-site intervention can be targeted to treat the abnormal finding, thus decreasing the number of supplementary examinations required and also reducing the likelihood of redundant interventions. We did not measure the cost of treatment. However, it follows that a reduction in resource use would also reduce costs.

Our study suggests an association between ICU and in-hospital mortalities and structured use of POCUS during ward emergencies although these findings were not confirmed after matching. To the best of our knowledge, no RCT has shown such an association, and certainly not on the ward. One multicenter, prospective, controlled study conducted in hypotensive patients in the emergency department showed no association between the use of POCUS and patient outcomes [34]. However, the treatment provided was similar in the two arms, suggesting that benefit from the use of POCUS was not maximalized. At the same time, the study was stopped prematurely because the treating physicians felt it was unethical to resuscitate patients without the use of POCUS. Conversely, two studies suggested an association between the use of ultrasound and patient outcomes. In a before–after study, Kanji et al. found that limited echocardiography-guided therapy was associated with a reduction in mortality in patients with shock [35]. Patients who were treated based on echocardiography findings received less intravenous fluids. In a retrospective analysis of a multicenter database, Feng et al. also showed that the use of echocardiography in septic patients was associated with reduced mortality. However, treatments based on echocardiography findings resulted in more fluids in the first 24 h [36]. Regardless of the amount of fluids transfused, the findings in these studies are similar to ours with regards to the association with improved outcomes.

Ray et al. showed that inappropriate diagnosis and treatment of patients in acute respiratory failure are associated with increased mortality [37]. We observed earlier intervention in the POCUS group, as did others [7]. The lower mortality rates observed with the use of POCUS may stem from earlier intervention. POCUS use reduces diagnostic uncertainty [26] and can identify life-threatening conditions that would otherwise be missed [38]. The use of a handheld POCUS device has also been shown to be particularly efficient for cardiac diagnosis [39–42]. In the current study, the reduced use of invasive mechanical ventilation, steroids and vasopressors in the POCUS group suggests more targeted treatment in patients with an underlying cardiac cause for deterioration. In our study, the proportion of appropriate intervention at the bedside was similar in the two groups but the number of interventions was significantly higher in the control group than in the POCUS group. Thus, POCUS makes it possible to adjust early the best treatment.

Our study has several limitations. It is a single-center study, which limits the extrapolation of our findings. However, the in-hospital mortality of our control group (35%) is similar to that described in other studies and aligns with the predicted mortality of our cohort [43, 44]. Our study was only controlled. Although the two groups were well balanced, the patients in the control group may have been sicker (as evidenced by their lower MAP, higher mottling scores and higher oxygen flows), and regarding the outcomes, our results were not significant after matching. However, we adjusted the mortality rates to account for this potential bias and our results remained significant in most of the models chosen. There was no crossover between the two RRT. The probability of a specific team effect is reduced by the fact that during the study period a large number of seniors and juniors randomly composed each RRT. We focused our study on the first treatment administered by the RRT and not on treatment during the first 24 h, which may have differed in the two groups. However, our patients were managed in the same institution, by similar ward physicians, surgeons and intensivists. Finally, the contribution of POCUS cannot be separated from that of the actual treatment protocol in our study. Regardless, the use of a combined strategy seems to be associated with improved performance.

## Conclusion

Our clinical trial suggests that protocolized use of a handheld POCUS device at the bedside in the ward by RRT may improve the proportion of adequate diagnosis, the time to initial treatment and perhaps also survival of ward patients developing acute respiratory and/or circulatory failure. However, because the control group was slightly sicker than the POCUS group, our results need to be confirmed by future multicenter, randomized, controlled trials.

## Supplementary Information

**Additional file 1.** **Additional Table 1**: Glossary of terms used in the study (supplement material).

**Additional file 2.** **Additional Table 2**: List of evocated diagnosis (A) and care management (B) after management at the bedside in ward (supplement material).

**Additional file 3.** **Additional Table 3**: Summary of patients’ characteristics (supplement material).

**Additional file 4.** **Additional Method**: Details of propensity score (supplement material).

**Additional file 5.** **Additional Table 4**: Summary of senior and resident ICU physicians medical experience between the two groups (supplement material).

**Additional file 6.** **Additional Table 5**: Propensity score between the two groups (supplement material).

**Additional file 7.** **Additional Table 6**: POCUS effect group on in-hospital mortality (logistic regressions, sensitivity analyses) (supplement material).

## Data Availability

All the data generated and analyzed during this study are included in this published article.

## Abbreviations

CI: Confidence interval
HHUD: Handheld ultrasound devices
ICU: Intensive care unit
IQR: Interquartile range
MAP: Mean arterial pressure
OR: Odds ratio
POCUS: Point-of-care ultrasound
RRT: Rapid response team
SAPS: Simplified Acute Physiology Score
