# Supplementary material for: Bedside POCUS during ward emergencies is associated with improved diagnosis and outcome: an observational, prospective, controlled study
Source: Crit Care. 2021 Jan 22;25:34. doi: 10.1186/s13054-021-03466-z (PMC7825196; doi:10.1186/s13054-021-03466-z)
Supplement: Supplementary file 1 — Additional file 1. Additional Table 1: Glossary of terms used in the study (supplement material). [file 13054_2021_3466_MOESM1_ESM.docx]

**Online additional data**

**Bedside POCUS during ward emergencies is associated with improved diagnosis and outcome: An observational prospective controlled study.**

Laurent Zieleskiewicz, MD, PhD^1,6^ (0000-0002-0788-4967), Alexandre Lopez, MD^1^, Sami Hraiech, MD, PhD^2^, Karine Baumstarck, MD, PhD^3^, Bruno Pastene, MD^1^, Mathieu Di Bisceglie, MD^4^, Benjamin Coiffard, MD^2^, Gary Duclos, MD^1^, Alain Boussuges, MD, PhD^5,6^, Xavier Bobbia, MD, PhD^7^, Sharon Einav, MD^8^, Laurent Papazian, MD, PhD^2^, Marc Leone, MD, PhD^1^

^1^ Aix Marseille University, Assistance Publique Hôpitaux de Marseille, Department of Anaesthesiology and Intensive Care, Hôpital Nord, Marseille, 13015, France. ^2^ Aix Marseille University, Assistance Publique Hôpitaux de Marseille, Service de Médecine Intensive ‑ Réanimation, Hôpital Nord, Marseille, 13015, France. ^3^ Centre d'Etudes et de Recherches sur les Services de Santé et Qualité, Faculté de Médecine, Aix-Marseille Université, Marseille, 13005, France. ^4^ Aix Marseille University, Assistance Publique Hôpitaux de Marseille, Service d'Imagerie Médicale, Hôpital Nord, Marseille, 13015, France. ^5^ Aix Marseille University, Assistance Publique Hôpitaux de Marseille, Service des Explorations Fonctionnelles Respiratoires, Marseille, 13015, France. ^6^ Center for Cardiovascular and Nutrition Research (C2VN) Aix Marseille Université, INSERM, INRA, Marseille, 13005, France. ^7^ Department of Anaesthesiology, Emergency and Critical Care Medicine, Intensive Care Unit, Nîmes, 30000, University Hospital Nîmes France. ^8^ Surgical Intensive Care Unit, Shaare Zedek Medical Center and Hebrew University Faculty of Medicine, Jerusalem, Israel.

**Additional Table 1: Glossary of terms used in the study**

| **Categories of variables** |  | **Definitions** |
| --- | --- | --- |
| *Introduction* | Rapid Response Team (RRT) | In our institution, the RRT is composed of an ICU physician senior, an ICU resident and a medical student. The equipment includes a resuscitation backpack, a transport monitor (EKG, spo2, NIBP, CO2) and a transport ventilator.  In case of in-hospital emergency suspicion, the physician (senior or resident) or the nurse in charge in the ward can call the RRT. |
|  | Acute respiratory failure | Syndrome refers to a inadequate gas exchange by the respiratory system with an hypoxemia and/or hypercapnia. It is due to an abnormality of the chest wall, lung parenchyma, respiratory muscles, airways, central nervous system. In our institution, symptoms of acute respiratory failure may be : hypoxemia (pulse oximetry <90%), sweats, rapid breathing (>25 breaths a minute), an inability to breathe (<8 breaths a minute), bluish coloration of skin, sleepiness, obstructed airway, noisy breathing or stridor.  The acute respiratory failure was determinated by the physician in charge in ward. |
|  | Acute circulatory failure | Syndrome refers to an inadequate perfusion of organs with metabolic and cellular disorders. It’s mainly associated with tachycardia, hypotension and signs of hypoperfusion. It can be due to a cardiogenic, hypovolemic or hemorrhagic, obstructive shock.  In our institution, circulatory failure was defined by a bradycardia (pulse < 40 beats a minute), a tachycardia (pulse > 120 beats a minute), a low blood pressure (median < 65 mmHg) or a urine output < 50ml over four hours. The circulatory failure was determinated by the physician in charge in ward. |
| *Methods* | Immediate diagnosis in ward | Diagnosis at the bedside after assessment with or without a handheld ultrasound device by the RRT. |
|  | Time to immediate diagnosis | Time between the arrival of the RRT and the first diagnosis at the bedside in ward after examination with or without the hand-held ultrasound device. |
|  | Time to first treatment | Time between the arrival of the RRT and the first administrated treatment at the bedside in ward after examination with or without the hand-held ultrasound device. |
|  | Orientation | Immediat ICU : Immediat admission in Intensive care unit of the hospital.  Operative room : for surgery.  Emergency room : in our hospital, in the Emergency department, there is a place for the intra-hospital emergencies in case of out of immediate capacity in ICU.  If referred to the emergency room or the operative room, after care management critically ill patients were admitted in ICU.  In ward if the management is possible. |
| *Results* | Appropriate intervention or treatment in ward | The good administrated treatment or care management in case of the good immediate diagnosis in ward by the RRT in charge. |
|  | Number of administrated treatments or intervention | All treatments performed during the initial care management of the patient at the bedside by the RRT. |
|  | Number of supplementary exams during first day | All supplementary exams performed during the initial management of the patients and permitted the final diagnosis. |
|  | ICU length of stay | Length of stay in ICU if case of admission in ICU. |
|  | Hospital length of stay | Length of stay in hospital of the patients between the admission and the out of the hospital. |
| *Abbreviations:* RRT, Rapid Response Team; EKG, electrocardiogram; spo2, pulse oximetry; NIBP, non-invasive bloop pressure; CO2, capnogram | | |
