# Supplementary material for: Bedside POCUS during ward emergencies is associated with improved diagnosis and outcome: an observational, prospective, controlled study
Source: Crit Care. 2021 Jan 22;25:34. doi: 10.1186/s13054-021-03466-z (PMC7825196; doi:10.1186/s13054-021-03466-z)
Supplement: Supplementary file 2 — Additional file 2. Additional Table 2: List of evocated diagnosis (A) and care management (B) after management at the bedside in ward (supplement material). [file 13054_2021_3466_MOESM2_ESM.docx]

**Online additional data**

**Bedside POCUS during ward emergencies is associated with improved diagnosis and outcome: An observational prospective controlled study.**

Laurent Zieleskiewicz, MD, PhD^1,6^ (0000-0002-0788-4967), Alexandre Lopez, MD^1^, Sami Hraiech, MD, PhD^2^, Karine Baumstarck, MD, PhD^3^, Bruno Pastene, MD^1^, Mathieu Di Bisceglie, MD^4^, Benjamin Coiffard, MD^2^, Gary Duclos, MD^1^, Alain Boussuges, MD, PhD^5,6^, Xavier Bobbia, MD, PhD^7^, Sharon Einav, MD^8^, Laurent Papazian, MD, PhD^2^, Marc Leone, MD, PhD^1^

^1^ Aix Marseille University, Assistance Publique Hôpitaux de Marseille, Department of Anaesthesiology and Intensive Care, Hôpital Nord, Marseille, 13015, France. ^2^ Aix Marseille University, Assistance Publique Hôpitaux de Marseille, Service de Médecine Intensive ‑ Réanimation, Hôpital Nord, Marseille, 13015, France. ^3^ Centre d'Etudes et de Recherches sur les Services de Santé et Qualité, Faculté de Médecine, Aix-Marseille Université, Marseille, 13005, France. ^4^ Aix Marseille University, Assistance Publique Hôpitaux de Marseille, Service d'Imagerie Médicale, Hôpital Nord, Marseille, 13015, France. ^5^ Aix Marseille University, Assistance Publique Hôpitaux de Marseille, Service des Explorations Fonctionnelles Respiratoires, Marseille, 13015, France. ^6^ Center for Cardiovascular and Nutrition Research (C2VN) Aix Marseille Université, INSERM, INRA, Marseille, 13005, France. ^7^ Department of Anaesthesiology, Emergency and Critical Care Medicine, Intensive Care Unit, Nîmes, 30000, University Hospital Nîmes France. ^8^ Surgical Intensive Care Unit, Shaare Zedek Medical Center and Hebrew University Faculty of Medicine, Jerusalem, Israel.

**Additional Table 2: List of evocated diagnosis (A) and care management (B) after management at the bedside in ward**

In each group, after screening and inclusion of patients, the physician had to make the hypothesis of a diagnosis at the bedside.
This list was previously established by the physicians of the RRT. The list was similar in the 2 groups.

**(A)**

| **Respiratory failure - Calling reason** | **Circulatory failure – Calling reason** |
| --- | --- |
| Pneumothorax | Septic shock |
| Pleural effusion | Hypovolemic shock |
| Pneumonia | Hemorrhagic shock |
| Atelectasis | Cardiogenic shock |
| Acute interstitial lung disease (non-cardiogenic edema) | Pulmonary embolism |
| Cardiogenic edema | Tamponade / Acute right heart failure |
| Exacerbation of Asthma | Pneumothorax |
| Exacerbation of COPD  Pulmonary embolism | Pleural effusion  Normal |
| Normal | Other |
| Other |  |

**(B)**

| **Diagnosis** | **Care management** |
| --- | --- |
| Pneumothorax | Drainage, volemic expansion, oxygen therapy |
| Pleural effusion | Drainage, volemic expansion, oxygen therapy |
| Pneumonia | Antibiotherapy, oxygen therapy |
| Atelectasia | Physiotherapy, oxygen therapy |
| Acute interstitial lung disease (non-cardiogenic edema) | Oxygen therapy or invasive ventilation |
| Cardiogenic edema | Diuretics, nitrates, noninvasive ventilation, oxygen therapy |
| Exacerbation of asthma | Aerosols bronchodilatators, aerosols corticoids, systemics corticoids, oxygen therapy |
| Exacerbation of COPD | Aerosols bronchodilatators, aerosols corticoids, systemics corticoids, antibiotics, noninvasive ventilation |
| Pulmonary embolism | Curative anticoagulation, oxygen therapy |
| Septic shock | Antibiotics, volemic expansion, vasopressors |
| Hypovolemic shock | Volemic expansion, vasopressors |
| Hemorrhagic shock | Transfusion, volemic expansion, vasopressors |
| Tamponade | Drainage, volemic expansion, operative room, vasopressors |
