# Supplementary material for: Bedside POCUS during ward emergencies is associated with improved diagnosis and outcome: an observational, prospective, controlled study
Source: Crit Care. 2021 Jan 22;25:34. doi: 10.1186/s13054-021-03466-z (PMC7825196; doi:10.1186/s13054-021-03466-z)
Supplement: Supplementary file 3 — Additional file 3. Additional Table 3: Summary of patients’ characteristics (supplement material). [file 13054_2021_3466_MOESM3_ESM.docx]

**Online additional data**

**Bedside POCUS during ward emergencies is associated with improved diagnosis and outcome: An observational prospective controlled study.**

Laurent Zieleskiewicz, MD, PhD^1,6^ (0000-0002-0788-4967), Alexandre Lopez, MD^1^, Sami Hraiech, MD, PhD^2^, Karine Baumstarck, MD, PhD^3^, Bruno Pastene, MD^1^, Mathieu Di Bisceglie, MD^4^, Benjamin Coiffard, MD^2^, Gary Duclos, MD^1^, Alain Boussuges, MD, PhD^5,6^, Xavier Bobbia, MD, PhD^7^, Sharon Einav, MD^8^, Laurent Papazian, MD, PhD^2^, Marc Leone, MD, PhD^1^

^1^ Aix Marseille University, Assistance Publique Hôpitaux de Marseille, Department of Anaesthesiology and Intensive Care, Hôpital Nord, Marseille, 13015, France. ^2^ Aix Marseille University, Assistance Publique Hôpitaux de Marseille, Service de Médecine Intensive ‑ Réanimation, Hôpital Nord, Marseille, 13015, France. ^3^ Centre d'Etudes et de Recherches sur les Services de Santé et Qualité, Faculté de Médecine, Aix-Marseille Université, Marseille, 13005, France. ^4^ Aix Marseille University, Assistance Publique Hôpitaux de Marseille, Service d'Imagerie Médicale, Hôpital Nord, Marseille, 13015, France. ^5^ Aix Marseille University, Assistance Publique Hôpitaux de Marseille, Service des Explorations Fonctionnelles Respiratoires, Marseille, 13015, France. ^6^ Center for Cardiovascular and Nutrition Research (C2VN) Aix Marseille Université, INSERM, INRA, Marseille, 13005, France. ^7^ Department of Anaesthesiology, Emergency and Critical Care Medicine, Intensive Care Unit, Nîmes, 30000, University Hospital Nîmes France. ^8^ Surgical Intensive Care Unit, Shaare Zedek Medical Center and Hebrew University Faculty of Medicine, Jerusalem, Israel.

**Additional Table 3: Summary of patients’ characteristics**

1. **Control group**

*Abbréviations :* ICU, Intensive care unit; COPD, Chronic obstructive pulmonary disease; CT scan, Computerized tomography scan.

| **N°** | **Sex** | **Age** | **SAPS II** | **Indication for placing the call to the RRT** | **Immediate diagnosis at bedside** | **Orientation** | **Definitive diagnosis** | **Means to definitive diagnosis** | **Adequate**  **diagnosis** | **In-Hospital Mortality** |
| --- | --- | --- | --- | --- | --- | --- | --- | --- | --- | --- |
| 1 | Woman | 53 | 42 | Respiratory failure | Asthma / COPD | In ward | Asthma | Medical files / Xray | Yes | Yes |
| 2 | Woman | 49 | 19 | Respiratory failure | Pneumothorax | ICU | Pneumonia | Medical files / CT scan - Xray - endoscopy | No | No |
| 3 | Woman | 81 | 49 | Respiratory failure | Cardiogenic edema | ICU | Cardiogenic edema | Medical files / Xray - comprehensive echocardiography | Yes | Yes |
| 4 | Man | 58 | 24 | Respiratory failure | Cardiogenic edema | ICU | Cardiogenic edema | Medical files / Xray - comprehensive echocardiography | Yes | No |
| 5 | Man | 48 | 19 | Respiratory failure | Pneumonia | ICU | Pneumonia | Medical files / CT scan - Xray - endoscopy | Yes | No |
| 6 | Man | 63 | 57 | Respiratory failure | Pneumonia | ICU | Pneumonia | Medical files / CT scan - Xray - endoscopy | Yes | Yes |
| 7 | Man | 68 | 18 | Respiratory failure | Asthma / COPD | ICU | COPD / pneumothorax | Medical files / CT scan - Xray | Yes | Yes |
| 8 | Man | 76 | 34 | Respiratory failure | Asthma / COPD | Emergency room | Cardiogenic edema | Medical files / comprehensive echocardiography | No | No |
| 9 | Woman | 48 | 50 | Respiratory failure | Pulmonary embolism | ICU | Valve thrombosis | Medical files / CT scan | No | No |
| 10 | Man | 60 | 54 | Circulatory failure | Septic shock | ICU | Septic shock | Medical files / CT scan - comprehensive echocardiography - bacteriological test | Yes | No |
| 11 | Woman | 73 | 43 | Respiratory failure | Pneumonia | ICU | Pneumonia | Medical files / CT - endoscopy | Yes | Yes |
| 12 | Man | 62 | 27 | Circulatory failure | Hypovolemic / hemorrhagic shock | ICU | Hypovolemic shock | Medical files / CT scan | Yes | No |
| 13 | Woman | 71 | 49 | Respiratory failure | Asthma / COPD | ICU | Cardiogenic edema | Medical files / Xray - comprehensive echocardiography | No | No |
| 14 | Man | 54 | 43 | Circulatory failure | Cardiogenic shock | ICU | Cardiogenic shock | Medical files / Xray - comprehensive echocardiography | Yes | No |
| 15 | Woman | 64 | 42 | Respiratory failure | Pneumonia | ICU | Pneumonia | Medical files / CT - endoscopy - bacteriological test | Yes | No |
| 16 | Man | 86 | 48 | Circulatory failure | Septic shock | ICU | Septic shock | Medical files / CT scan - comprehensive echocardiography - bacteriological test | Yes | No |
| 17 | Man | 51 | 59 | Circulatory failure | Hypovolemic / hemorrhagic shock | ICU | Pleural effusion | Medical files / CT scan - comprehensive echocardiography | No | Yes |
| 18 | Man | 75 | 62 | Respiratory failure | Pneumonia | ICU | Acute interstitial lung disease (non-cardiogenic edema) | Medical files / CT scan - Xray - endoscopy | No | Yes |
| 19 | Woman | 60 | 22 | Respiratory failure | Pneumonia | ICU | Atelectasia | Medical files / Xray | No | Yes |
| 20 | Man | 63 | 33 | Respiratory failure | Asthma / COPD | ICU | COPD | Medical files / CT scan - Xray | Yes | No |
| 21 | Woman | 59 | 33 | Respiratory failure | Pneumonia | In ward | Pneumonia | Medical files / CT scan - Xray - bacteriological test | Yes | No |
| 22 | Woman | 91 | 40 | Circulatory failure | Septic shock | ICU | Septic shock | Medical files / CT scan - comprehensive echocardiography - bacteriological test | Yes | Yes |
| 23 | Man | 63 | 35 | Respiratory failure | Pneumonia | In ward | Pneumonia | Medical files / CT scan - bacteriological test | Yes | No |
| 24 | Woman | 79 | 70 | Circulatory failure | Septic shock | ICU | Septic shock | Medical files / CT scan - bacteriological test | Yes | No |
| 25 | Man | 53 | 25 | Respiratory failure | Asthma / COPD | ICU | COPD | Medical files / CT scan - Xray - endoscopy | Yes | No |
| 26 | Man | 79 | 53 | Respiratory failure | Pneumonia | ICU | Pneumonia / Acute interstitial lung disease (non-cardiogenic edema) | Medical files / CT - endoscopy - bacteriological test | Yes | No |
| 27 | Man | 55 | 33 | Respiratory failure | Asthma / COPD | Emergency room | COPD / pneumonia | Medical files / CT - endoscopy - bacteriological test | Yes | No |
| 28 | Woman | 81 | 44 | Respiratory failure | Pulmonary embolism | ICU | Pulmonary embolism | Medical files / CT scan - comprehensive echocardiography | Yes | No |
| 29 | Man | 84 | 65 | Circulatory failure | Tamponade | ICU | Tamponade | Medical files / comprehensive echocardiography | Yes | Yes |
| 30 | Woman | 77 | 38 | Circulatory failure | Septic shock | ICU | Septic shock | Medical files / CT scan - comprehensive echocardiography - bacteriological test | Yes | No |
| 31 | Man | 23 | 9 | Respiratory failure | Other | ICU | Other / anaphylaxis | Medical files / biology | Yes | No |
| 32 | Man | 79 | 53 | Circulatory failure | Hypovolemic / hemorrhagic shock | Operative room | Hemorrhagic shock | Medical files / CT scan | Yes | No |
| 33 | Man | 57 | 22 | Respiratory failure | Pneumonia | ICU | Pneumonia | Medical files / CT scan - comprehensive echocardiography | Yes | No |
| 34 | Man | 77 | 58 | Circulatory failure | Septic shock | Emergency room | Hemorragic shock | Medical files / CT scan - Xray - bacteriological test | Yes | No |
| 35 | Man | 77 | 30 | Respiratory failure | Pneumonia | ICU | Pneumonia | Medical files / CT scan - Xray - bacteriological test | Yes | No |
| 36 | Woman | 82 | 49 | Respiratory failure | Cardiogenic edema | Emergency room | Cardiogenic edema | Medical files / CT scan - Xray - bacteriological test | Yes | Yes |
| 37 | Woman | 55 | 18 | Respiratory failure | Asthma / COPD | ICU | COPD | Medical files / CT scan - Xray - bacteriological test | Yes | No |
| 38 | Woman | 78 | 98 | Circulatory failure | Hypovolemic / hemorrhagic shock | ICU | Hemorrhagic shock | Medical files / CT scan - comprehensive echocardiography | Yes | Yes |
| 39 | Woman | 75 | 51 | Respiratory failure | Cardiogenic edema | ICU | Cardiogenic edema | Medical files / Xray - comprehensive echocardiography | Yes | No |
| 40 | Man | 87 | 62 | Circulatory failure | Septic shock | In ward | Septic shock | Medical files / Xray - bacteriological test | Yes | Yes |
| 41 | Man | 88 | 48 | Respiratory failure | Pulmonary embolism | ICU | Pulmonary embolism | Medical files / CT scan | Yes | No |
| 42 | Man | 62 | 31 | Circulatory failure | Septic shock | ICU | Septic shock | Medical files / CT scan - comprehensive echocardiography - bacteriological test | Yes | No |
| 43 | Man | 64 | 90 | Circulatory failure | Cardiogenic shock | ICU | Septic shock | Medical files / CT scan - coronary angiography | No | Yes |
| 44 | Man | 69 | 46 | Respiratory failure | Asthma / COPD | ICU | Asthma | Medical files / CT scan - Xray - comprehensive echocardiography | Yes | No |
| 45 | Man | 70 | 46 | Respiratory failure | Pneumonia | ICU | Acute interstitial lung disease (non-cardiogenic edema) | Medical files / CT scan - Xray - endoscopy | No | Yes |
| 46 | Woman | 92 | 60 | Respiratory failure | Pneumonia | In ward | Pneumonia | Medical files / Xray - comprehensive echocardiography | Yes | Yes |
| 47 | Man | 65 | 27 | Respiratory failure | Other | In ward | Other / tracheal decanulation | Medical files | Yes | No |
| 48 | Woman | 84 | 59 | Circulatory failure | Hypovolemic / hemorrhagic shock | ICU | Hemorrhagic shock | Medical files / CT scan | Yes | No |
| 49 | Woman | 76 | 30 | Respiratory failure | Cardiogenic edema | In ward | Cardiogenic edema | Medical files / Xray | Yes | No |
| 50 | Man | 86 | 44 | Respiratory failure | Pulmonary embolism | In ward | Pleural effusion | Medical files / CT scan - Xray - comprehensive echocardiography | No | No |
| 51 | Man | 68 | 18 | Circulatory failure | Hypovolemic / hemorrhagic shock | ICU | Hemorrhagic shock | Medical files / CT scan | Yes | No |
| 52 | Woman | 90 | 48 | Respiratory failure | Cardiogenic edema | ICU | Cardiogenic edema | Medical files / CT scan - comprehensive echocardiography | Yes | Yes |
| 53 | Woman | 24 | 45 | Respiratory failure | Pneumothorax | ICU | Pneumothorax | Medical files / CT scan - Xray | Yes | No |
| 54 | Man | 58 | 72 | Respiratory failure | Pneumonia | ICU | Acute interstitial lung disease (non-cardiogenic edema) | Medical files / CT scan - Xray - comprehensive echocardiography | No | Yes |
| 55 | Woman | 68 | 51 | Respiratory failure | Other | Operative room | Other / bronchospasm | Medical files / Xray - endoscopy | Yes | No |
| 56 | Man | 70 | 63 | Respiratory failure | Pneumonia | ICU | Pneumonia | Medical files / CT - endoscopy | Yes | No |
| 57 | Woman | 52 | 49 | Respiratory failure | Other | ICU | Other / intraalveolar hemorrhage | Medical files / CT - endoscopy | Yes | Yes |
| 58 | Man | 85 | 61 | Respiratory failure | Pneumonia | ICU | Pneumonia | Medical files / CT scan - Xray - bacteriological test | Yes | No |
| 59 | Man | 73 | 68 | Circulatory failure | Septic shock | Operative room | Septic shock | Medical files / CT scan - comprehensive echocardiography | Yes | Yes |
| 60 | Man | 54 | 27 | Circulatory failure | Septic shock | In ward | Septic shock | Medical files / CT scan - bacteriological test | Yes | No |
| 61 | Woman | 67 | 37 | Respiratory failure | Pneumonia | ICU | Pneumonia | Medical files / CT scan - Xray | Yes | No |
| 62 | Man | 57 | 54 | Respiratory failure | Pneumonia | ICU | Pneumonia | Medical files / CT scan - Xray - endoscopy | Yes | No |
| 63 | Man | 52 | 34 | Respiratory failure | Asthma / COPD | ICU | Asthma | Medical files / CT scan | Yes | No |
| 64 | Man | 70 | 71 | Circulatory failure | Septic shock | ICU | Septic shock | Medical files / CT scan | Yes | Yes |
| 65 | Man | 57 | 48 | Respiratory failure | Pneumonia | ICU | COPD | Medical files / CT scan | No | No |
| 66 | Woman | 54 | 75 | Circulatory failure | Septic shock | ICU | Septic shock | Medical files / CT scan - comprehensive echocardiography - endoscopy | Yes | Yes |
| 67 | Man | 60 | 35 | Respiratory failure | Asthma / COPD | In ward | COPD / pneumothorax | Medical files / CT scan - Xray | Yes | No |
| 68 | Man | 76 | 44 | Respiratory failure | Pneumonia | ICU | Pleural effusion | Medical files / CT scan - Xray - comprehensive echocardiography | No | No |
| 69 | Woman | 86 | 57 | Circulatory failure | Septic shock | In ward | Septic shock | Medical files / CT scan | Yes | Yes |
| 70 | Man | 72 | 46 | Respiratory failure | Asthma / COPD | In ward | Cardiogenic edema | Medical files / comprehensive echocardiography | No | No |
| 71 | Woman | 27 | 35 | Respiratory failure | Pneumonia | In ward | Pneumonia | Medical files / CT scan - comprehensive echocardiography - endoscopy | Yes | Yes |
| 72 | Man | 61 | 49 | Circulatory failure | Hypovolemic / hemorrhagic shock | ICU | Hemorrhagic shock | Medical files / CT scan - comprehensive echocardiography | Yes | No |
| 73 | Man | 55 | 60 | Respiratory failure | Pneumonia | ICU | Pneumonia | Medical files / CT scan - comprehensive echocardiography - endoscopy | Yes | Yes |
| 74 | Woman | 87 | 46 | Respiratory failure | Cardiogenic edema | ICU | Cardiogenic edema | Medical files / Xray - comprehensive echocardiography | Yes | No |
| 75 | Man | 69 | 49 | Circulatory failure | Septic shock | ICU | Septic shock | Medical files / CT scan - comprehensive echocardiography | Yes | Yes |
| 76 | Woman | 66 | 75 | Respiratory failure | Asthma / COPD | ICU | Neurological failure | Medical files / CT scan - comprehensive echocardiography | No | Yes |
| 77 | Woman | 77 | 33 | Circulatory failure | Hypovolemic / hemorrhagic shock | ICU | Hemorrhagic shock | Medical files / CT scan | Yes | No |
| 78 | Woman | 29 | 56 | Respiratory failure | Acute interstitial lung disease (non-cardiogenic edema) | Operative room | Acute interstitial lung disease (non-cardiogenic edema) | Medical files / CT scan - comprehensive echocardiography | Yes | No |
| 79 | Woman | 51 | 35 | Respiratory failure | Asthma / COPD | In ward | Other / pulmonary hypertension | Medical files / CT scan - comprehensive echocardiography - right catheterization | No | No |
| 80 | Woman | 67 | 37 | Respiratory failure | Asthma / COPD | ICU | Pneumonia | Medical files / CT scan - endoscopy | No | No |
| 81 | Man | 76 | 60 | Respiratory failure | Pneumonia | ICU | Pneumonia | Medical files / CT scan - comprehensive echocardiography | Yes | Yes |
| 82 | Man | 61 | 35 | Circulatory failure | Septic shock | Operative room | Cardiogenic shock | Medical files / CT scan - comprehensive echocardiography | No | Yes |

1. **POCUS group**

| **N°** | **Sex** | **Age** | **SAPS II** | **Indication for placing the call to the RRT** | **Immediate diagnosis at bedside** | **Orientation** | **Definitive diagnosis** | **Means to definitive diagnosis** | **Adequate diagnosis** | **In-Hospital Mortality** |
| --- | --- | --- | --- | --- | --- | --- | --- | --- | --- | --- |
| 1 | Man | 56 | 24 | Circulatory failure | Septic shock | Operative room | Septic shock | Medical files / operative room | Yes | No |
| 2 | Man | 33 | 48 | Respiratory failure | Pneumonia | Icu | Pneumonia | Medical files / CT scan - endoscopy | Yes | Yes |
| 3 | Man | 72 | 67 | Circulatory failure | Septic shock | Icu | Septic shock | Medical files / CT scan - bacteriological test | Yes | No |
| 4 | Man | 68 | 63 | Respiratory failure | Cardiogenic edema | Icu | Cardiogenic edema | Medical files / comprehensive echocardiography | Yes | No |
| 5 | Man | 69 | 62 | Respiratory failure | Pulmonary embolism | Icu | Pulmonary embolism | Medical files / CT scan | Yes | No |
| 6 | Woman | 65 | 48 | Respiratory failure | Other | Icu | Other / anaphylaxia | Medical files / biology | Yes | No |
| 7 | Man | 55 | 55 | Respiratory failure | Acute interstitial lung disease (non-cardiogenic edema) | Icu | Acute interstitial lung disease (non-cardiogenic edema) | Medical files / CT scan | Yes | No |
| 8 | Man | 67 | 40 | Respiratory failure | Cardiogenic edema | Icu | Cardiogenic edema | Medical files / CT scan - Xray | Yes | No |
| 9 | Man | 69 | 72 | Circulatory failure | Septic shock | Icu | Septic shock | Medical files / CT scan - Xray | Yes | No |
| 10 | Man | 71 | 40 | Respiratory failure | Pneumonia | Icu | Pneumonia / Cardiogenic edema | Medical files / CT scan - Xray | Yes | No |
| 11 | Woman | 82 | 39 | Circulatory failure | Cardiogenic shock | Icu | Cardiogenic shock | Medical files / comprehensive echocardiography - Xray | Yes | No |
| 12 | Woman | 72 | 61 | Respiratory failure | Atelectasia | Icu | COPD | Medical files / Xray | No | No |
| 13 | Woman | 21 | 23 | Respiratory failure | Pneumonia | Icu | Pneumonia | Medical files / Xray | Yes | No |
| 14 | Man | 84 | 107 | Respiratory failure | Acute interstitial lung disease (non-cardiogenic edema) | Icu | Acute interstitial lung disease (non-cardiogenic edema) | Medical files / CT scan | Yes | Yes |
| 15 | Man | 82 | 37 | Respiratory failure | Cardiogenic edema | Icu | Cardiogenic edema | Medical files / comprehensive echocardiography | Yes | No |
| 16 | Woman | 89 | 52 | Circulatory failure | Septic shock | Operative room | Septic shock | Medical files / CT scan - endoscopy - bacteriological test | Yes | Yes |
| 17 | Man | 36 | 26 | Respiratory failure | Normal | Icu | Normal | Medical files / Xray | Yes | No |
| 18 | Man | 64 | 45 | Respiratory failure | Pneumonia | Icu | Pneumonia | Medical files / CT scan | Yes | No |
| 19 | Woman | 78 | 40 | Respiratory failure | Acute interstitial lung disease (non-cardiogenic edema) | Icu | Acute interstitial lung disease (non-cardiogenic edema) / pneumonia | Medical files / CT scan - Xray | Yes | No |
| 20 | Man | 85 | 66 | Circulatory failure | Pleural effusion | Icu | Septic shock | Medical files / CT scan - Xray | No | No |
| 21 | Man | 83 | 24 | Respiratory failure | Normal | In ward | Normal / panic attack | Medical files / CT scan | Yes | No |
| 22 | Woman | 37 | 27 | Circulatory failure | Septic shock | Icu | Septic shock | Medical files / CT scan - endoscopy - bacteriological test | Yes | No |
| 23 | Woman | 56 | 30 | Circulatory failure | Septic shock | Operative room | Septic shock | Medical files / CT scan - bacteriological test | Yes | No |
| 24 | Woman | 50 | 48 | Respiratory failure | Other | Icu | Other / laryngeal dyspnea | Medical files / endoscopy | Yes | No |
| 25 | Man | 44 | 44 | Respiratory failure | Cardiogenic edema | Icu | Cardiogenic edema | Medical files / CT scan - endoscopy - Xray | Yes | No |
| 26 | Woman | 90 | 65 | Respiratory failure | Cardiogenic edema | In ward | Cardiogenic edema | Medical files / CT scan - comprehensive echocardiography | Yes | No |
| 27 | Man | 70 | 45 | Respiratory failure | Cardiogenic edema | Icu | Cardiogenic edema | Medical files / comprehensive echocardiography | Yes | No |
| 28 | Woman | 67 | 45 | Respiratory failure | Cardiogenic edema | Icu | Cardiogenic edema | Medical files / Xray | Yes | No |
| 29 | Woman | 84 | 38 | Respiratory failure | Asthma / COPD | Emergency room | Asthma | Medical files / CT scan - Xray | Yes | No |
| 30 | Woman | 87 | 35 | Respiratory failure | Atelectasia | In ward | Pneumonia | Medical files / Xray | No | Yes |
| 31 | Man | 78 | 34 | Circulatory failure | Septic shock | In ward | Septic shock | Medical files / CT scan - endoscopy - bacteriological test | Yes | No |
| 32 | Man | 67 | 25 | Circulatory failure | Tamponade | Operative room | Tamponade | Medical files / comprehensive echocardiography | Yes | No |
| 33 | Woman | 96 | 39 | Respiratory failure | Cardiogenic edema | Icu | Cardiogenic edema | Medical files / comprehensive echocardiography | Yes | No |
| 34 | Woman | 81 | 56 | Respiratory failure | Pneumonia | Emergency room | Pneumonia | Medical files / CT scan - Xray | Yes | No |
| 35 | Woman | 66 | 58 | Respiratory failure | Atelectasia | Icu | Atelectasia | Medical files / CT scan - endoscopy | Yes | Yes |
| 36 | Man | 71 | 21 | Respiratory failure | Asthma / COPD | In ward | COPD | Medical files | Yes | No |
| 37 | Man | 65 | 37 | Circulatory failure | Hypovolemic / hemorrhagic shock | In ward | Hemorrhagic shock | Medical files | Yes | No |
| 38 | Man | 38 | 32 | Circulatory failure | Hypovolemic / hemorrhagic shock | Emergency room | Hemorrhagic shock | Medical files / CT scan | Yes | No |
| 39 | Man | 72 | 36 | Respiratory failure | Tamponade | Icu | Acute interstitial lung disease (non-cardiogenic edema) | Medical files / CT scan - comprehensive echocardiography | No | No |
| 40 | Man | 57 | 42 | Respiratory failure | Cardiogenic edema | In ward | Cardiogenic edema | Medical files / comprehensive echocardiography | Yes | No |
| 41 | Man | 62 | 51 | Respiratory failure | Acute interstitial lung disease (non-cardiogenic edema) | Icu | Acute interstitial lung disease (non-cardiogenic edema) | Medical files / CT scan - comprehensive echocardiography - Xray | Yes | Yes |
| 42 | Woman | 76 | 36 | Respiratory failure | Acute interstitial lung disease (non-cardiogenic edema) | Emergency room | Acute interstitial lung disease (non-cardiogenic edema) | Medical files / CT scan | Yes | No |
| 43 | Man | 70 | 32 | Respiratory failure | Cardiogenic edema | Icu | Cardiogenic edema | Medical files / comprehensive echocardiography - Xray | Yes | No |
| 44 | Woman | 42 | 59 | Circulatory failure | Septic shock | In ward | Septic shock | Medical files | Yes | No |
| 45 | Man | 61 | 45 | Respiratory failure | Asthma / COPD | Icu | COPD | Medical files / comprehensive echocardiography - Xray | Yes | No |
| 46 | Man | 87 | 58 | Respiratory failure | Asthma / COPD | Emergency room | COPD | Medical files / CT scan - Xray | Yes | No |
| 47 | Man | 83 | 83 | Circulatory failure | Hypovolemic / hemorrhagic shock | Icu | Hemorrhagic shock | Medical files / CT scan | Yes | No |
| 48 | Man | 63 | 33 | Respiratory failure | Other | In ward | Other / hemoptysis | Medical files / CT scan - endoscopy - Xray | Yes | No |
| 49 | Man | 90 | 43 | Respiratory failure | Cardiogenic edema | Emergency room | Cardiogenic edema | Medical files / comprehensive echocardiography | Yes | Yes |
| 50 | Man | 59 | 58 | Respiratory failure | Pneumonia | Icu | Pneumonia | Medical files / CT scan - endoscopy | Yes | No |
| 51 | Man | 71 | 25 | Respiratory failure | Asthma / COPD | In ward | COPD | Medical files / CT scan - endoscopy | Yes | No |
| 52 | Woman | 70 | 40 | Respiratory failure | Pleural effusion | Icu | Pleural effusion | Medical files / CT scan - comprehensive echocardiography - Xray | Yes | Yes |
| 53 | Man | 69 | 27 | Respiratory failure | Atelectasia | Icu | Atelectasia | Medical files / Xray | Yes | No |
| 54 | Man | 49 | 30 | Respiratory failure | Asthma / COPD | Emergency room | COPD | Medical files / CT scan - Xray | Yes | No |
| 55 | Man | 81 | 37 | Respiratory failure | Pulmonary embolism | Icu | Pulmonary embolism | Medical files / CT scan - Xray | Yes | No |
| 56 | Man | 83 | 47 | Respiratory failure | Other | Icu | Neurological faillure | Medical files / CT scan | Yes | No |
| 57 | Woman | 80 | 74 | Circulatory failure | Septic shock | Icu | Septic shock | Medical files / comprehensive echocardiography - Xray | Yes | Yes |
| 58 | Man | 65 | 60 | Circulatory failure | Septic shock | Icu | Septic shock | Medical files / CT scan - bacteriological test | Yes | No |
| 59 | Man | 82 | 46 | Respiratory failure | Pneumonia | Icu | Pneumonia | Medical files / CT scan - Xray | Yes | No |
| 60 | Man | 84 | 57 | Circulatory failure | Septic shock | Operative room | Septic shock | Medical files / bacteriological test | Yes | No |
| 61 | Woman | 92 | 40 | Respiratory failure | Cardiogenic edema | Icu | Cardiogenic edema | Medical files / Xray | Yes | No |
| 62 | Man | 79 | 51 | Circulatory failure | Tamponade | Operative room | Tamponade | Medical files | Yes | Yes |
| 63 | Man | 76 | 35 | Respiratory failure | Cardiogenic edema | In ward | Cardiogenic edema / COPD | Medical files / comprehensive echocardiography | Yes | No |
| 64 | Woman | 55 | 34 | Respiratory failure | Pleural effusion | Icu | Pleural effusion | Medical files / Xray | Yes | No |
| 65 | Man | 45 | 49 | Respiratory failure | Pneumothorax | Icu | Pneumothorax | Medical files / CT scan - Xray | Yes | No |
| 66 | Woman | 80 | 40 | Respiratory failure | Cardiogenic edema | Emergency room | Cardiogenic edema | Medical files / CT scan - comprehensive echocardiography | Yes | No |
| 67 | Woman | 77 | 33 | Respiratory failure | Asthma / COPD | Icu | Asthma | Medical files / Xray | Yes | No |
| 68 | Man | 49 | 52 | Circulatory failure | Hypovolemic / hemorrhagic shock | Operative room | Hemorrhagic shock | Medical files / CT scan | Yes | No |
| 69 | Man | 72 | 52 | Respiratory failure | Atelectasia | Icu | Atelectasia | Medical files / CT scan - endoscopy | Yes | No |
| 70 | Man | 63 | 34 | Respiratory failure | Pneumonia | In ward | Pneumonia | Medical files / CT scan - bacteriological test | Yes | No |
| 71 | Man | 55 | 17 | Respiratory failure | Asthma / COPD | In ward | Asthma | Medical files | Yes | No |
| 72 | Man | 82 | 49 | Respiratory failure | Cardiogenic edema | Icu | Cardiogenic edema | Medical files / CT scan - Xray | Yes | No |
| 73 | Man | 76 | 57 | Respiratory failure | Acute interstitial lung disease (non-cardiogenic edema) | In ward | Acute interstitial lung disease (non-cardiogenic edema) | Medical files / CT scan - Xray | Yes | Yes |
| 74 | Man | 82 | 67 | Respiratory failure | Acute interstitial lung disease (non-cardiogenic edema) | Icu | Acute interstitial lung disease (non-cardiogenic edema) / pneumonia | Medical files / CT scan - endoscopy | Yes | Yes |
| 75 | Woman | 74 | 36 | Circulatory failure | Septic shock | Emergency room | Septic shock | Medical files / CT scan - endoscopy - Xray | Yes | No |
| 76 | Man | 61 | 35 | Circulatory failure | Septic shock | In ward | Septic shock | Medical files / CT scan - Xray | Yes | No |
| 77 | Woman | 36 | 26 | Respiratory failure | Pleural effusion | In ward | Pleural effusion | Medical files / CT scan | Yes | Yes |
| 78 | Woman | 77 | 30 | Respiratory failure | Atelectasia | In ward | Atelectasia | Medical files / Xray | Yes | No |
| 79 | Man | 70 | 40 | Respiratory failure | Pleural effusion | In ward | Pleural effusion | Medical files / Xray | Yes | Yes |
| 80 | Man | 70 | 43 | Respiratory failure | Atelectasia | In ward | Other / morphine overdosage | Medical files / CT scan | No | No |
| 81 | Woman | 58 | 32 | Respiratory failure | Normal | In ward | Normal | Medical files | Yes | No |
| 82 | Man | 36 | 27 | Circulatory failure | Septic shock | In ward | Septic shock | Medical files | Yes | No |
| 83 | Man | 84 | 27 | Respiratory failure | Acute interstitial lung disease (non-cardiogenic edema) | Icu | Pneumonia / Acute interstitial lung disease (non-cardiogenic edema) | Medical files / CT scan - endoscopy | Yes | No |
