# Supplementary material for: Bedside POCUS during ward emergencies is associated with improved diagnosis and outcome: an observational, prospective, controlled study
Source: Crit Care. 2021 Jan 22;25:34. doi: 10.1186/s13054-021-03466-z (PMC7825196; doi:10.1186/s13054-021-03466-z)
Supplement: Supplementary file 4 — Additional file 4. Additional Method: Details of propensity score (supplement material). [file 13054_2021_3466_MOESM4_ESM.docx]

**Online additional data**

**Bedside POCUS during ward emergencies is associated with improved diagnosis and outcome: An observational prospective controlled study.**

Laurent Zieleskiewicz, MD, PhD^1,6^ (0000-0002-0788-4967), Alexandre Lopez, MD^1^, Sami Hraiech, MD, PhD^2^, Karine Baumstarck, MD, PhD^3^, Bruno Pastene, MD^1^, Mathieu Di Bisceglie, MD^4^, Benjamin Coiffard, MD^2^, Gary Duclos, MD^1^, Alain Boussuges, MD, PhD^5,6^, Xavier Bobbia, MD, PhD^7^, Sharon Einav, MD^8^, Laurent Papazian, MD, PhD^2^, Marc Leone, MD, PhD^1^

^1^ Aix Marseille University, Assistance Publique Hôpitaux de Marseille, Department of Anaesthesiology and Intensive Care, Hôpital Nord, Marseille, 13015, France. ^2^ Aix Marseille University, Assistance Publique Hôpitaux de Marseille, Service de Médecine Intensive ‑ Réanimation, Hôpital Nord, Marseille, 13015, France. ^3^ Centre d'Etudes et de Recherches sur les Services de Santé et Qualité, Faculté de Médecine, Aix-Marseille Université, Marseille, 13005, France. ^4^ Aix Marseille University, Assistance Publique Hôpitaux de Marseille, Service d'Imagerie Médicale, Hôpital Nord, Marseille, 13015, France. ^5^ Aix Marseille University, Assistance Publique Hôpitaux de Marseille, Service des Explorations Fonctionnelles Respiratoires, Marseille, 13015, France. ^6^ Center for Cardiovascular and Nutrition Research (C2VN) Aix Marseille Université, INSERM, INRA, Marseille, 13005, France. ^7^ Department of Anaesthesiology, Emergency and Critical Care Medicine, Intensive Care Unit, Nîmes, 30000, University Hospital Nîmes France. ^8^ Surgical Intensive Care Unit, Shaare Zedek Medical Center and Hebrew University Faculty of Medicine, Jerusalem, Israel.

**Additional Method: Details of propensity score**

1. **Selection of variables to be included in the propensity score model**

The definitive choice was based on both logistic regression (T) and clinical significance (P). A total of 8 variables, true confounders (T) and predictors (P) of the outcome (adequate diagnosis), are included in the propensity score model: SAPS II (P), mottling’s presence (P), respiratory rate (T), pulse oximetry (T), need for oxygen therapy (P), chronic heart failure (P), chronic obstructive pulmonary disease (T), and sex (P). The definitive choice of the 8 variables was validated by 3 authors including two ICU senior physicians (LZ and ML) and the methodologist of the study (KB).

1. **Matching method**

The matching was performed using the R MatchIt package (version 3.5.3 (2018-07-02); Feather Spray Copyright (C) 2018 The R Foundation for Statistical Computing) and was based on the nearest neighbor with-caliper matching (means only matching a control case and a POCUS case if they are within 0.05 standard deviations of propensity score away from each other).

Code: m.out2 = matchit(*covariates* , data = data.frame(mat2), method = "nearest", distance = "logit", replace = T, caliper = 0.05).

1. **Accuracy of the propensity score**

- Histograms of propensity scores before and after matching:

| **Before matching** | **After matching** |
| --- | --- |
| 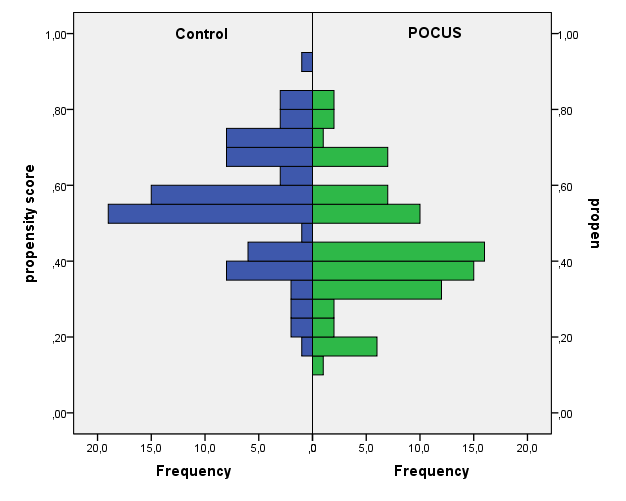 | 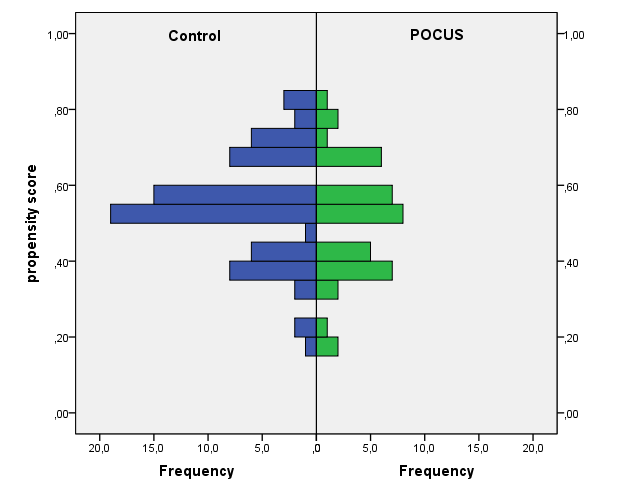 |

- Standardized mean differences (SMD) of the different covariates after matching:

| **Variable** | **Control** | **POCUS** | **SMD (95% CI)** |
| --- | --- | --- | --- |
| SAPS II | 43.8 ± 16.8 | 44.0 ± 15.8 | 0.01 [-0.37 - 0.39] |
| Mottling’s presence | 0.21 | 0.22 | 0.01 [-0.37 - 0.39] |
| Respiratory rate | 26.9 ± 8.4 | 27.8 ± 8.3 | 0.10 [-0.27 - 0.49] |
| Pulse oxymetry | 91.6 ± 7.1 | 92.0 ± 6.8 | 0.05 [-0.32 - 0.44] |
| Need for oxygen therapy | 0.91 | 0.95 | 0.15 [-0.23 - 0.53] |
| Chronic heart failure | 0.29 | 0.22 | 0.15 [-0.23 - 0.53] |
| Chronic obstructive pulmonary disease | 0.43 | 0.44 | 0.02 [-0.36 - 0.40] |
| Sex | 0.62 | 0.58 | 0.09 [-0.29 - 0.47] |
| *Abbreviations:* SAPS, Simplified acute physiology score ; CI, confidence interval | | | |
