# Supplementary material for: Bedside POCUS during ward emergencies is associated with improved diagnosis and outcome: an observational, prospective, controlled study
Source: Crit Care. 2021 Jan 22;25:34. doi: 10.1186/s13054-021-03466-z (PMC7825196; doi:10.1186/s13054-021-03466-z)
Supplement: Supplementary file 5 — Additional file 5. Additional Table 4: Summary of senior and resident ICU physicians medical experience between the two groups (supplement material). [file 13054_2021_3466_MOESM5_ESM.docx]

**Online additional data**

**Bedside POCUS during ward emergencies is associated with improved diagnosis and outcome: An observational prospective controlled study.**

Laurent Zieleskiewicz, MD, PhD^1,6^ (0000-0002-0788-4967), Alexandre Lopez, MD^1^, Sami Hraiech, MD, PhD^2^, Karine Baumstarck, MD, PhD^3^, Bruno Pastene, MD^1^, Mathieu Di Bisceglie, MD^4^, Benjamin Coiffard, MD^2^, Gary Duclos, MD^1^, Alain Boussuges, MD, PhD^5,6^, Xavier Bobbia, MD, PhD^7^, Sharon Einav, MD^8^, Laurent Papazian, MD, PhD^2^, Marc Leone, MD, PhD^1^

^1^ Aix Marseille University, Assistance Publique Hôpitaux de Marseille, Department of Anaesthesiology and Intensive Care, Hôpital Nord, Marseille, 13015, France. ^2^ Aix Marseille University, Assistance Publique Hôpitaux de Marseille, Service de Médecine Intensive ‑ Réanimation, Hôpital Nord, Marseille, 13015, France. ^3^ Centre d'Etudes et de Recherches sur les Services de Santé et Qualité, Faculté de Médecine, Aix-Marseille Université, Marseille, 13005, France. ^4^ Aix Marseille University, Assistance Publique Hôpitaux de Marseille, Service d'Imagerie Médicale, Hôpital Nord, Marseille, 13015, France. ^5^ Aix Marseille University, Assistance Publique Hôpitaux de Marseille, Service des Explorations Fonctionnelles Respiratoires, Marseille, 13015, France. ^6^ Center for Cardiovascular and Nutrition Research (C2VN) Aix Marseille Université, INSERM, INRA, Marseille, 13005, France. ^7^ Department of Anaesthesiology, Emergency and Critical Care Medicine, Intensive Care Unit, Nîmes, 30000, University Hospital Nîmes France. ^8^ Surgical Intensive Care Unit, Shaare Zedek Medical Center and Hebrew University Faculty of Medicine, Jerusalem, Israel.

|  | | **Control group**  **n = 10** | **POCUS group**  **n = 16** | **P value** |
| --- | --- | --- | --- | --- |
| **Senior physicians** | |  |  |  |
| Medical formation,  n (%) | Anesthesiologist-Intensivist | 7 | 16 |  |
|  | Intensivist-Pneumologist | 2 | 0 |  |
|  | Intensivist-Nephrologist | 1 | 0 |  |
| Medical experience, mean ± SD, years | | 8 ± 3 | 5 ± 2 | 0.59 |
| Medical experience < 2 years, n % | | 4 | 8 |  |
| Medical experience 2 - 5 years, n % | | 0 | 3 |  |
| Medical experience > 5 years, n % | | 6 | 5 |  |
|  | | **Control group**  **n = 31** | **POCUS group**  **n = 32** | **P value** |
| **Junior Physicians** | |  |  |  |
| Medical experience, mean ± SD, years | | 2.7 ± 0.3 | 2.6 ± 0.6 | 0.89 |

**Additional Table 4: Summary of senior and resident ICU physicians medical experience between the two groups**
