# Supplementary material for: Bedside POCUS during ward emergencies is associated with improved diagnosis and outcome: an observational, prospective, controlled study
Source: Crit Care. 2021 Jan 22;25:34. doi: 10.1186/s13054-021-03466-z (PMC7825196; doi:10.1186/s13054-021-03466-z)
Supplement: Supplementary file 6 — Additional file 6. Additional Table 5: Propensity score between the two groups (supplement material). [file 13054_2021_3466_MOESM6_ESM.docx]

**Online additional data**

**Bedside POCUS during ward emergencies is associated with improved diagnosis and outcome: An observational prospective controlled study.**

Laurent Zieleskiewicz, MD, PhD^1,6^ (0000-0002-0788-4967), Alexandre Lopez, MD^1^, Sami Hraiech, MD, PhD^2^, Karine Baumstarck, MD, PhD^3^, Bruno Pastene, MD^1^, Mathieu Di Bisceglie, MD^4^, Benjamin Coiffard, MD^2^, Gary Duclos, MD^1^, Alain Boussuges, MD, PhD^5,6^, Xavier Bobbia, MD, PhD^7^, Sharon Einav, MD^8^, Laurent Papazian, MD, PhD^2^, Marc Leone, MD, PhD^1^

^1^ Aix Marseille University, Assistance Publique Hôpitaux de Marseille, Department of Anaesthesiology and Intensive Care, Hôpital Nord, Marseille, 13015, France. ^2^ Aix Marseille University, Assistance Publique Hôpitaux de Marseille, Service de Médecine Intensive ‑ Réanimation, Hôpital Nord, Marseille, 13015, France. ^3^ Centre d'Etudes et de Recherches sur les Services de Santé et Qualité, Faculté de Médecine, Aix-Marseille Université, Marseille, 13005, France. ^4^ Aix Marseille University, Assistance Publique Hôpitaux de Marseille, Service d'Imagerie Médicale, Hôpital Nord, Marseille, 13015, France. ^5^ Aix Marseille University, Assistance Publique Hôpitaux de Marseille, Service des Explorations Fonctionnelles Respiratoires, Marseille, 13015, France. ^6^ Center for Cardiovascular and Nutrition Research (C2VN) Aix Marseille Université, INSERM, INRA, Marseille, 13005, France. ^7^ Department of Anaesthesiology, Emergency and Critical Care Medicine, Intensive Care Unit, Nîmes, 30000, University Hospital Nîmes France. ^8^ Surgical Intensive Care Unit, Shaare Zedek Medical Center and Hebrew University Faculty of Medicine, Jerusalem, Israel.

**Additional Table 5: Propensity score between the two groups**

Eight covariates were included in the propensity score model (SAPS 2 score, mottling, respiratory rate, oxygen saturation, need for oxygen therapy, chronic heart failure, chronic respiratory failure, and sex). The matching identified 42 POCUS and 73 control individuals.

**(A) Primary, secondary outcomes of patients**^a^

|  | | **Matched cohort** | | |
| --- | --- | --- | --- | --- |
| **Outcome category** | | **Control group**  **n = 73** | **POCUS group**  **n = 42** | **P-value** |
| **Primary outcome** | |  |  |  |
| **Immediate adequate diagnosis** | **General** | **57 (78)** | **39 (93)** | **0.04** |
|  | Circulatory | 20/22 (91) | 11/11 (100) | 0.30 |
|  | Respiratory | 37/51 (72) | 28 /31 (90) | 0.05 |
| **Secondary outcomes** | |  |  |  |
| Appropriate  intervention in ward | General | 63 (86) | 41 (98) | 0.04 |
|  | Circulatory | 18/22 (82) | 11/11 (100) | 0.13 |
|  | Respiratory | 43/51 (84) | 30/31 (97) | 0.08 |
| Time to immediate diagnosis  Median [IQR], min | General | 15 [5 - 20] | 10 [5 - 15] | 0.06 |
|  | Circulatory | 10 [7 - 20] | 10 [5 - 18] | 0.51 |
|  | Respiratory | 15 [5 - 20] | 9 [5 -15] | 0.07 |
| Time to first treatment / intervention  Median [IQR], min | General | 30 [15 - 40] | 15 [10 - 30] | 0.001 |
|  | Circulatory | 28 [15 - 45] | 15 [10 - 40] | 0.10 |
|  | Respiratory | 30 [17 - 40] | 15 [10 - 30] | 0.002 |
| Number of interventions, median [IQR] | General | 4 [3 - 4] | 3 [2 - 4] | 0.005 |
|  | Circulatory | 4 [3 - 4] | 3 [2 - 4] | 0.06 |
|  | Respiratory | 3 [2 -4] | 3 [2 -4] | 0.03 |
| Number of supplementary exams during first day, median [IQR] | General | 2 [1 - 3] | 1 [1 - 2] | 0.00002 |
|  | Circulatory | 2 [2 - 3] | 1 [0 - 2] | 0.12 |
|  | Respiratory | 2 [1 -3] | 1 [1 - 2] | 0.001 |
| ICU length of stay, median [IQR], days | General | 5 [2 - 10] | 2 [1 - 6] | 0.04 |
|  | Circulatory | 3 [2 - 6] | 2 [1 - 4] | 0.56 |
|  | Respiratory | 5 [3 - 12] | 3 [1 - 6] | 0.06 |
| Hospital length of stay, median [IQR], days | General | 16 [9 - 29] | 18 [7 - 30] | 0.77 |
|  | Circulatory | 13 [8 - 28] | 10 [6 - 38] | 0.56 |
|  | Respiratory | 17 [10 - 29] | 18 [7 - 29] | 0.63 |
| *Abbreviations:* IQR: Interquartile range  ^a^Data are expressed as No (%) of participants unless otherwise indicated. | | | | |

**(B) Administrated treatments by the Rapid Response Teams at the bedside in the POCUS versus the physician judgement (control) treatment groups** ^a^

|  | **Matched cohort^b^** | | |
| --- | --- | --- | --- |
|  | **Control group**  **n = 73** | **POCUS group**  **n = 42** | **P-value** |
| Drainage | 5 (7) | 6 (14) | 0.19 |
| Volemic expansion | 32 (44) | 15 (36) | 0.39 |
| Diuretics | 13 (18) | 7 (17) | 0.87 |
| Nitrates | 2 (3) | 3 (7) | 0.27 |
| Noninvasive ventilation | 15 (21) | 8 (19) | 0.84 |
| Oxygen therapy | 54 (74) | 28 (67) | 0.40 |
| Invasive ventilation | 14 (19) | 4 (9) | 0.17 |
| Aerosol bronchodilatators | 22 (30) | 11 (26) | 0.65 |
| Aerosol corticoids | 9 (12) | 3 (7) | 0.38 |
| Systemic corticoid | 13 (18) | 4 (9) | 0.23 |
| Antibiotic | 38 (51) | 14 (33) | 0.05 |
| Curative anticoagulation | 4 (5) | 4 (9) | 0.41 |
| Aspirin | 2 (3) | 0 | 0.28 |
| Inotrop | 2 (3) | 3 (7) | 0.27 |
| Vasoppressor | 21 (29) | 2 (5) | 0.002 |
| Antiarythmic | 1 (1) | 0 | 0.44 |
| Transfusion | 4 (5) | 1 (2) | 0.44 |
| Physiotherapy | 7 (10) | 6 (14) | 0.44 |
| ^a^Data are expressed as No (%) of participants | | | |

**(C) In-hospital and ICU mortality rates in the POCUS versus the physician judgement treatment groups**

|  |  | **Matched cohort^a^** | | |
| --- | --- | --- | --- | --- |
|  |  | **Control group**  **n = 73** | **POCUS group**  **n = 42** | **P-value** |
| In-ICU mortality  **Ligne 95** | General | * 13 (22) | * 7 (25) | 0.79 |
|  | Circulatory |  |  |  |
|  | Respiratory |  |  |  |
| **In-Hospital mortality** | **General** | **25 (34)** | **12 (29)** | **0.53** |
|  | Circulatory | 10/22 (46) | 2/11 (18) | 0.11 |
|  | Respiratory | 15/51 (29) | 10/31 (32) | 0.78 |
| * Patients admitted in ICU: Control group n =58 and POCUS group n = 28 | | | | |
