# Supplementary material for: Bedside POCUS during ward emergencies is associated with improved diagnosis and outcome: an observational, prospective, controlled study
Source: Crit Care. 2021 Jan 22;25:34. doi: 10.1186/s13054-021-03466-z (PMC7825196; doi:10.1186/s13054-021-03466-z)
Supplement: Supplementary file 7 — Additional file 7. Additional Table 6: POCUS effect group on in-hospital mortality (logistic regressions, sensitivity analyses) (supplement material). [file 13054_2021_3466_MOESM7_ESM.docx]

**Online additional data**

**Bedside POCUS during ward emergencies is associated with improved diagnosis and outcome: An observational prospective controlled study.**

Laurent Zieleskiewicz, MD, PhD^1,6^ (0000-0002-0788-4967), Alexandre Lopez, MD^1^, Sami Hraiech, MD, PhD^2^, Karine Baumstarck, MD, PhD^3^, Bruno Pastene, MD^1^, Mathieu Di Bisceglie, MD^4^, Benjamin Coiffard, MD^2^, Gary Duclos, MD^1^, Alain Boussuges, MD, PhD^5,6^, Xavier Bobbia, MD, PhD^7^, Sharon Einav, MD^8^, Laurent Papazian, MD, PhD^2^, Marc Leone, MD, PhD^1^

^1^ Aix Marseille University, Assistance Publique Hôpitaux de Marseille, Department of Anaesthesiology and Intensive Care, Hôpital Nord, Marseille, 13015, France. ^2^ Aix Marseille University, Assistance Publique Hôpitaux de Marseille, Service de Médecine Intensive ‑ Réanimation, Hôpital Nord, Marseille, 13015, France. ^3^ Centre d'Etudes et de Recherches sur les Services de Santé et Qualité, Faculté de Médecine, Aix-Marseille Université, Marseille, 13005, France. ^4^ Aix Marseille University, Assistance Publique Hôpitaux de Marseille, Service d'Imagerie Médicale, Hôpital Nord, Marseille, 13015, France. ^5^ Aix Marseille University, Assistance Publique Hôpitaux de Marseille, Service des Explorations Fonctionnelles Respiratoires, Marseille, 13015, France. ^6^ Center for Cardiovascular and Nutrition Research (C2VN) Aix Marseille Université, INSERM, INRA, Marseille, 13005, France. ^7^ Department of Anaesthesiology, Emergency and Critical Care Medicine, Intensive Care Unit, Nîmes, 30000, University Hospital Nîmes France. ^8^ Surgical Intensive Care Unit, Shaare Zedek Medical Center and Hebrew University Faculty of Medicine, Jerusalem, Israel.

**Additional Table 6: POCUS effect group on in-hospital mortality (logistic regressions, sensitivity analyses)**

| Adjustment for: | | OR [95% CI] | p-value |
| --- | --- | --- | --- |
| 1 | age, sex | 0.36 [0.17-0.76] | 0.007 |
| 2 | age, sex, serum lactate* | 0.32 [0.14-0.72] | 0.006 |
| 3 | age, sex, serum lactate*, MAP° | 0.35 [0.16-0.79] | 0.011 |
| 4 | age, sex, serum lactate*, MAP°, oxygen need | 0.39 [0.17-0.89] | 0.025 |
| 5 | age, sex, serum lactate*, MAP°, oxygen need, SAPS II | 0.41 [0.17-0.95] | 0.038 |
| 6 | age, sex, serum lactate*, MAP°, oxygen need, SAPS II, ICU admission | 0.40 [0.17-0.94] | 0.035 |
| 7 | sex, serum lactate*, serum creatinine, SAPS II, ROX index (1)(2) | 0.48 [0.20-1.12] | 0.089 |
| 8 | age, serum creatinine, SAPS II, ICU admission, ROX index | 0.48 [0.21-1.11] | 0.084 |
| 9 | age, mottling, MAP° | 0.45 [0.20-0.95] | 0.035 |
| 10 | age, mottling, MAP°, serum lactate* | 0.36 [0.16-0.83] | 0.016 |
| 11 | age, sex, mottling, MAP°, serum lactate*, oxygen need | 0.39 [0.17-0.89] | 0.025 |
| 12 | Serum creatinine, oxygen need, SAPS II, ICU admission, ROX index | 0.48 [0.21-1.11] | 0.085 |
| 13 | age, mottling, serum lactate*, MAP°, oxygen rate flow^#^, N° of treatments | 0.36 [0.15-0.87] | 0.023 |
| * mmol/l; ° mmHg ; ^#^ l/min  Abbreviation: MAP: Mean arterial pressure ; SAPS: Simplified acute physiology score ; ICU: intensive care unit | | | |

**References**

1. Roca O, Messika J, Caralt B, García-de-Acilu M, Sztrymf B, Ricard J-D, et al. Predicting success of high-flow nasal cannula in pneumonia patients with hypoxemic respiratory failure: The utility of the ROX index. J Crit Care. 2016 Oct;35:200–5.

2. Frat J-P, Thille AW, Mercat A, Girault C, Ragot S, Perbet S, et al. High-flow oxygen through nasal cannula in acute hypoxemic respiratory failure. N Engl J Med. 2015 Jun 4;372(23):2185–96.
